# Single-cell bacterial transcription measurements reveal the importance of dimethylsulfoniopropionate (DMSP) hotspots in ocean sulfur cycling

**DOI:** 10.1038/s41467-020-15693-z

**Published:** 2020-04-23

**Authors:** Cherry Gao, Vicente I. Fernandez, Kang Soo Lee, Simona Fenizia, Georg Pohnert, Justin R. Seymour, Jean-Baptiste Raina, Roman Stocker

**Affiliations:** 1 Department of Biological Engineering, Massachusetts Institute of Technology, Cambridge, MA 02139 USA; 2 Department of Civil and Environmental Engineering, Ralph M. Parsons Laboratory, Massachusetts Institute of Technology, Cambridge, MA 02139 USA; 3 Department of Civil, Environmental and Geomatic Engineering, Institute for Environmental Engineering, ETH Zurich, 8093 Zurich, Switzerland; 4 Friedrich Schiller University, Institute of Inorganic and Analytical Chemistry, Jena, D-07743 Germany; 5 Climate Change Cluster (C3), University of Technology Sydney, Ultimo, NSW 2007 Australia

**Keywords:** Reporter genes, Marine microbiology, Biogeochemistry, Microbial ecology

## Abstract

Dimethylsulfoniopropionate (DMSP) is a pivotal compound in marine biogeochemical cycles and a key chemical currency in microbial interactions. Marine bacteria transform DMSP via two competing pathways with considerably different biogeochemical implications: demethylation channels sulfur into the microbial food web, whereas cleavage releases sulfur into the atmosphere. Here, we present single-cell measurements of the expression of these two pathways using engineered fluorescent reporter strains of *Ruegeria pomeroyi* DSS-3, and find that external DMSP concentration dictates the relative expression of the two pathways. DMSP induces an upregulation of both pathways, but only at high concentrations (>1 μM for demethylation; >35 nM for cleavage), characteristic of microscale hotspots such as the vicinity of phytoplankton cells. Co-incubations between DMSP-producing microalgae and bacteria revealed an increase in cleavage pathway expression close to the microalgae’s surface. These results indicate that bacterial utilization of microscale DMSP hotspots is an important determinant of the fate of sulfur in the ocean.

## Abstract (web summary)

DMSP is a ubiquitous organosulfur compound in the ocean that, once degraded by bacteria, plays key roles in global biogeochemical cycles and climate regulation. Here, the authors use single-cell measurements of transcription to investigate the intricate dynamics of bacterial DMSP degradation.

## Introduction

Up to 10% of the carbon fixed by phytoplankton cells in the ocean is converted to dimethylsulfoniopropionate (DMSP)^1^, resulting in a global production of this compound that exceeds one billion tons per year^2^. DMSP is an important currency in the ecological and metabolic exchanges between phytoplankton and heterotrophic bacteria^3^, as it represents a major nutrient source that contributes significantly to the sulfur and carbon demand of bacteria (up to 95% and 15%, respectively^4,5^). DMSP is utilized by marine bacteria via two competing catabolic pathways^6^: the demethylation pathway leads to the incorporation of both carbon and sulfur into bacterial biomass, whereas the cleavage pathway results in the utilization of carbon but the release of sulfur in the form of the climatically active gas dimethylsulfide (DMS). The environmental factors that govern the utilization of one pathway over the other, and ultimately the production and release of DMS to the atmosphere, have remained elusive, marking a major gap in the mechanistic link between microbial processes and global-scale carbon and sulfur biogeochemical cycles.

The water-column concentration of DMSP has been hypothesized to be an important factor regulating the choice of degradation pathway by bacteria (DMSP Availability Hypothesis^6^) and it has been speculated that bacteria control the fate of sulfur from DMSP by adjusting the relative expression of the demethylation and cleavage pathways (Bacterial Switch Hypothesis^7^). Concentrations of DMSP in bulk seawater are typically low, ranging from a few nanomolar (global oceanic average: 16.91 ± 22.17 nM^8^) up to 200 nM during phytoplankton blooms^9^. However, much higher DMSP concentrations are expected to occur in the vicinity of individual DMSP-producing organisms, such as phytoplankton cells, which can have intracellular DMSP concentrations of hundreds of millimolar^10^. Efforts to elucidate the environmental drivers of microbial catabolism of DMSP have to date been limited to measurements in large-volume batch cultures^11,12^ and seawater samples^13^. As a consequence, an understanding of the influence of microscale heterogeneity in DMSP concentrations on the microbial choice of degradation pathway is lacking.

Here, we report that the external concentrations of DMSP that are relevant for controlling the expression of degradation pathways by a model copiotrophic bacterium are unexpectedly high, and are characteristic of DMSP hotspots. This finding was enabled by the development of the first single-cell, time-resolved measurements of DMSP degradation pathway expression and their application to study the response of bacteria to different concentrations of DMSP.

## Results and discussion

### Construction and validation of fluorescent reporter bacteria

**Fig. 1:**
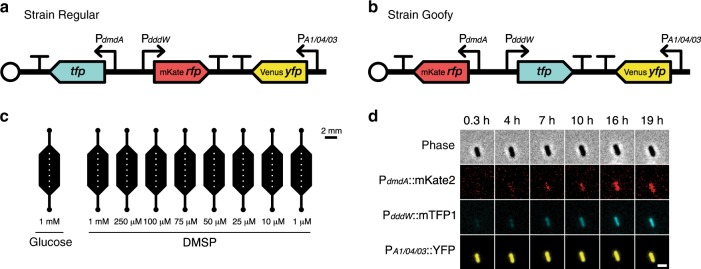
Single-cell measurements of DMSP degradation pathway expression. **a**, **b** Plasmids transformed into *R. pomeroyi* DSS-3 contain three components: *dmdA* reporter (222 bp promoter region); *dddW* reporter (500 bp promoter region); and constitutive *yfp* expression (strong, synthetic promoter P_*A1/04/03*_). YFP signal was used as a proxy for plasmid copy number and metabolic activity. Transcriptional terminators (represented by T) were placed between promoter fusion cassettes to prevent transcriptional read-through. To control for spectral bias caused by fluorescent protein choice (RFP or TFP), we constructed two *R. pomeroyi* reporter strains—Regular (**a**) and Goofy (**b**)—in which the colors of fluorescent proteins fused to *dmdA* and *dddW* promoter regions were interchanged. Vector backbone: pBBR1MCS-2 with origin of replication pBBR1 (open circles). **c** Schematic of a single microfluidic device used for time-lapse DMSP experiments. Each time-lapse DMSP experiment used one microfluidic device containing nine observation chambers for parallel incubation of a single reporter strain (Regular or Goofy; **a**, **b**) with different concentrations of DMSP. Glucose was used as negative control. White squares in each observation chamber represent the seven fields of view (200 μm × 200 μm) imaged at each time point. **d** Representative phase contrast and fluorescence images of a single cell (strain Goofy) over time in the presence of 1 mM DMSP. Scale bar, 2 μm.

To examine the relative expression of bacterial DMSP catabolism genes at the single-cell level, we genetically transformed *Ruegeria pomeroyi* DSS-3, a model Alphaproteobacterium from the Roseobacter clade. Like many members of the Roseobacter clade, which plays a central role in DMSP cycling^14^, *R. pomeroyi* harbors both DMSP catabolic pathways^15^. We transformed *R. pomeroyi* cells with a custom-built tricolor promoter-fusion plasmid designed to simultaneously report metabolic activity and the expression of the genes encoding the two DMSP degradation pathway enzymes through different fluorescence emission (Fig. 1a, b). In the engineered plasmid, the promoter regions of DMSP-dependent demethylase (DmdA) and DMSP lyase (DddW), which catalyze the first steps of the demethylation and cleavage pathways, respectively, control the expression of fluorescent proteins (Methods). Out of the three functional DMSP lyases (DddP, DddQ, and DddW)^16^ encoded in the *R. pomeroyi* DSS-3 genome, DddW was chosen in this study due to its strong upregulation response to DMSP reported in previous transcriptomic studies^11,17^, which suggests that it is the primary DMSP lyase in this bacterium. However, some of the cleavage dynamics, controlled by DddP and DddQ, may be missed by our approach.

Construction of promoter-fusion reporter strains, followed by quantitative single-cell time-lapse microscopy, has been commonly adopted to utilize fluorescence signal dynamics as a proxy for native gene expression behaviors^18–20^. To control for signal bias caused by the choice of fluorescent protein fused to each promoter region, we constructed two *R. pomeroyi* reporter strains (Goofy and Regular), for which we interchanged the color of fluorescent proteins fused to the *dmdA* and *dddW* promoter regions (Fig. 1a, b and Supplementary Fig. 1). The choice of fluorescent protein led to some differences in the temporal evolution of fluorescence signal, but did not affect our overall conclusions (Supplementary Fig. 2). A comparison of fluorescence signal output by tricolor and single-color reporter strains confirmed that promoter fusion cassettes induce fluorescent protein expression whether encoded alone or together (Supplementary Fig. 2).

To confirm that the strains specifically report *dmdA* and *dddW* gene expression, and to test for non-specific responses, the engineered bacteria were incubated with seven different carbon sources. Rich medium (5% 1/2 YTSS), propionate, acetate, succinate, and glucose did not elicit any fluorescence response (Supplementary Fig. 3). Glucose was chosen as the most suitable negative control for subsequent experiments for the following reasons: it elicited no non-specific DMSP gene transcription responses (Supplementary Fig. 3); its molecular weight is similar to DMSP; and the metabolic pathways of glucose and organosulfur compounds are distinct. The only carbon sources that led to an increase in cell fluorescence were DMSP (activating both *dmdA* and *dddW* promoters) and acrylate, a known *dmdA* inducer^21^ (activating only *dmdA*, but not *dddW*), thus confirming the validity of our reporter construct design (Supplementary Fig. 3).

### Time-lapse DMSP incubation experiments in microfluidic chips

**Fig. 2:**
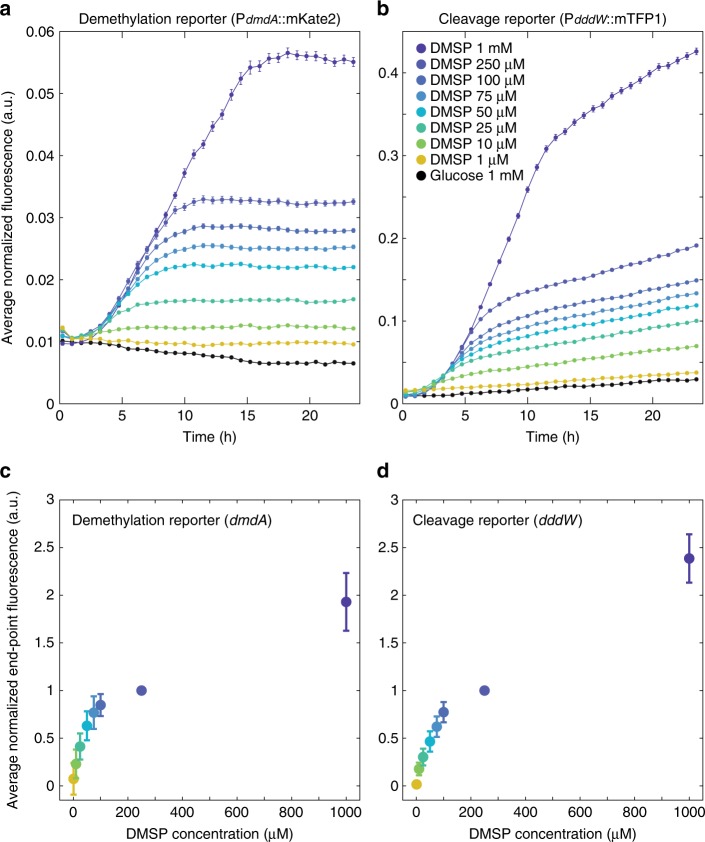
DMSP concentration-dependent upregulation of *dmdA* and *dddW*. **a**, **b** Mean fluorescence signals of *dmdA* (demethylation) (**a**) and *dddW* (cleavage) (**b**) reporters in response to different concentrations of DMSP. One representative replicate experiment of reporter strain Goofy is shown (data from additional replicate experiments (*n* = 3 for each reporter strain) are shown in Supplementary Fig. 6). Spectral leakage correction, background subtraction, and a threshold on YFP intensity were applied (see Methods and Supplementary Note 1). RFP and TFP signals of each cell were normalized by the mean YFP signal at each time point of each experimental condition. Data points and error bars represent means ± s.e.m. of cells (error bars may be smaller than markers). **c**, **d** Average end-point fluorescence levels of *dmdA* (**c**) and *dddW* (**d**) reporters. For each replicate experiment, baseline signal (glucose) was subtracted at each time point, fluorescence signals over the final five time points (~20.4–24 h) were averaged, then normalized by the corresponding end-point fluorescence signal at 250 μM DMSP. Data points and error bars represent means ± s.d. of six total replicates (*n* = 3 for strain Regular and *n* = 3 for strain Goofy combined).

A custom microfluidic chip containing nine observation chambers was employed for the simultaneous incubation of an engineered reporter strain with a range of concentrations of DMSP as a sole amended carbon source (Fig. 1c). The absence of fluid flow in the observation chambers enabled us to monitor the expression of DMSP degradation pathways in a time-resolved manner at the single-cell level (Fig. 1d). Images in phase contrast and fluorescence (in red, yellow, and teal channels) were acquired every 45 min for 24 h at six or seven positions per observation chamber (Fig. 2), encompassing 218 ± 120 (mean ± s.d.) cells per field of view (at *t* = 45 min) per condition (Supplementary Fig. 4). Microscope and camera settings were optimized to minimize phototoxicity and photobleaching while maximizing fluorescence signal capture.

**Fig. 3:**
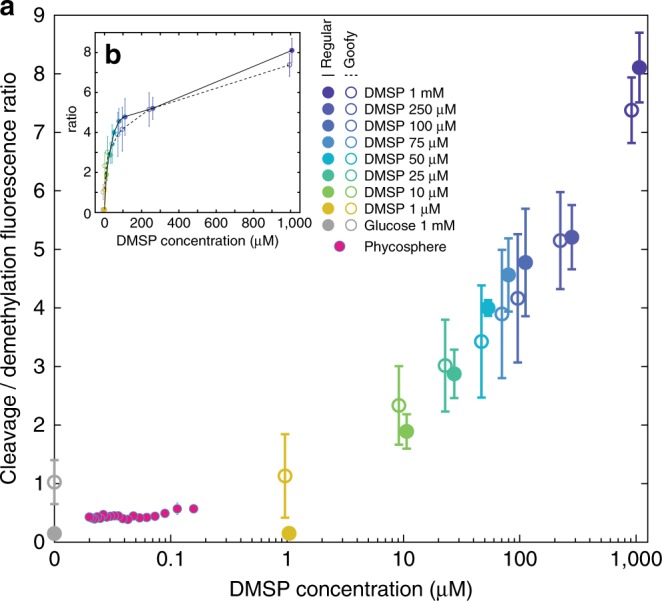
DMSP concentration modulates relative expression of *dddW* and *dmdA*. **a** Cleavage-to-demethylation pathway ratio was calculated at each DMSP concentration for strains Regular (RFP/TFP) and Goofy (TFP/RFP). High variability of fluorescence output amongst replicate experiments at ≥10 μM DMSP prevented the comparison of pathway reporters within each color (Supplementary Fig. 5). Average fluorescence signals at time points at which *dmdA* expression is mid-exponential for each DMSP concentration (shown in Supplementary Fig. 6), or at the second time point for glucose and 1 μM DMSP conditions, were used for ratio calculation. Close agreement between strains Regular and Goofy at ≥10 μM DMSP suggests that fluorescence ratios are close to true pathway expression ratios. The deviation between strains Regular and Goofy of ratios in glucose and 1 μM DMSP may be due to low fluorescence signals; importantly, ratios calculated within-color (0.3–0.5; Supplementary Fig. 5) and across-color (0.15–1.0; Fig. 3) in glucose showed consistent results (similar values at 1 μM DMSP). Pathway ratios from the phycosphere experiment (also shown in Fig. 4f) were calculated using TFP signals of reporter strains (reporting either *dmdA* or *dddW* expression in strains Regular or Goofy, respectively) normalized by constitutive YFP signals, and were plotted against modeled phycosphere DMSP concentrations (Supplementary Fig. 13). Data points and error bars of DMSP concentration experiments are slightly offset in the *x*-direction for presentation clarity, and represent means ± s.d. of replicate experiments (*n* = 3 for strain Regular; *n* = 3 for strain Goofy). Error bars of the phycosphere experiment represent the variance of the ratio of normalized cleavage and demethylation fluorescence signals (Supplementary Note 1). Error bars may be smaller than markers. **b** Inset represents the same data as **a**, plotted on a linear scale on the *x*-axis to show the saturating relationship between DMSP concentration and cleavage-to-demethylation ratio.

Low levels of expression of both pathways occurred even in the absence of DMSP (Supplementary Fig. 5 and Supplementary Note 1), with baseline *dmdA* expression 1.0–6.7 times higher than *dddW* expression (Fig. 3). High variability of fluorescence output among replicate experiments at ≥10 μM DMSP (Supplementary Fig. 6), likely caused by slight differences in subculture growth phase, prevented the comparison of pathway reporters within each color (Supplementary Fig. 5). Thus, across-color ratio calculation, which enabled comparisons of pathway expression within the same experiment, was employed in our study (Fig. 3). Importantly, the across-color ratios (0.15–1.0; Fig. 3) and within-color ratios (0.3–0.5; Supplementary Fig. 5) in glucose showed consistent results.

No significant upregulation beyond these baseline levels of either pathway was detected at DMSP concentrations below 1 μM (i.e., 100 nM and 500 nM) compared to negative controls (two-tailed *t*-tests, *n* = 259–2125 cells, *p* > 0.01) (Supplementary Fig. 7). At 1 μM DMSP, significant upregulation was only observed in some replicate experiments (Supplementary Fig. 8). Only one out of six replicate experiments of the demethylation pathway (*dmdA*) was upregulated at 1 μM DMSP, while the cleavage pathway (*dddW*) showed more consistent significant upregulation (four out of six replicate experiments) at 1 μM DMSP compared to glucose negative controls (two-tailed *t*-tests, *n* = 1284–9362 cells, *p* < 0.01) (Supplementary Fig. 8). At higher concentrations (≥10 μM), all replicate experiments exhibited upregulation (Supplementary Fig. 6). These results suggest that 1 μM approximates the threshold DMSP concentration above which bacteria start to increase *dmdA* gene expression beyond baseline levels. Consistent with existing evidence that points to demethylation as the major fate of DMSP in seawater^22^, our results also suggest that DMSP at typical bulk seawater concentrations (16.91 ± 22.17 nM^8^) is primarily degraded through the higher baseline expression levels of the demethylation pathway by Roseobacters (such as *R. pomeroyi*), which are major players in marine organic sulfur cycling^14^.

Both pathways were consistently and significantly upregulated compared to negative controls upon incubation with DMSP concentrations between 10 μM and 1 mM, which led to upregulation of 1.6–6.0-fold (*dmdA*) and 8.0–112.6-fold (*dddW*) compared to glucose at 24 h (Fig. 2a, b and Supplementary Figs. 6, 9; averages of *n* = 6 replicates). The rates of upregulation, expressed as the slope of the exponential phase of the fluorescence kinetics curves, were similar across different DMSP concentrations (10 μM–1 mM, Fig. 2a, b and Supplementary Fig. 6), suggesting that upon exposure to DMSP concentrations at or above 10 μM, cells are stimulated to initially increase the expression of both demethylation and cleavage pathways at a conserved rate.

While the rate of upregulation was conserved across DMSP concentrations, maximum gene expression levels of *dddW* and *dmdA* increased with DMSP concentration (Fig. 2c, d). Due to the stability of fluorescent proteins (half-lives of hours to more than a day^23^), the fluorescence signal is expected to persist even after gene expression returns to baseline levels. We therefore used the magnitude of the end-point fluorescence signal (averaged over the last five time points, i.e., 20.4–24 h) as a proxy for maximum gene expression levels of *dddW* and *dmdA*. Normalized maximum gene expression levels of both pathways increased approximately linearly with DMSP concentration between 1 and 75 μM DMSP (0.07–0.77 a.u. for *dmdA*; 0.02–0.62 a.u. for *dddW*; Fig. 2c, d). This increase plateaued above 100 μM (Fig. 2c, d), possibly as a result of the gene expression machinery becoming saturated and unable to respond as sensitively to DMSP at these high concentrations (Supplementary Note 2).

To determine how consumption of DMSP in the chambers may have affected our conclusions, we performed a larger-volume (8 ml) experiment in which we directly measured DMSP concentration and cell fluorescence, for selected timepoints (0, 2, 8, 24 h) and initial DMSP concentrations (1 μM, 75 μM, 1 mM). DMSP concentration decreased over time, due to uptake by bacteria (Supplementary Fig. 10). Consistent with results from the microfluidic chip experiments (Fig. 2), the initial rate of increase in the fluorescent signal was conserved between the 75 μM and 1 mM conditions, but decreased as the DMSP concentration diminished due to bacterial uptake (Supplementary Fig. 10). The saturation of the fluorescence signal coincided temporally with the depletion of DMSP (at 8 h, for the 75 μM condition; Supplementary Fig. 10). These results suggest that cells initially increase gene expression at a rate that is independent of DMSP concentration, but halt their gene upregulation when the DMSP supply is exhausted.

Rather than expressing only one pathway at any given DMSP concentration, as implied by the Bacterial Switch Hypothesis^7^, we observed that bacteria express both pathways simultaneously, but modulate the ratio of cleavage and demethylation according to DMSP concentration (Fig. 3). Overall, the cleavage-to-demethylation expression ratio increased with DMSP concentration up to 100 μM, above which it started to plateau (Fig. 3b). Baseline expression levels that are biased towards demethylation were represented by cleavage-to-demethylation ratios of 0.15–1.0 in the glucose negative controls (Fig. 3). At high DMSP concentrations between 10 μM and 1 mM, bacteria gradually skewed their gene expression towards the cleavage pathway, with the cleavage-to-demethylation ratio increasing from 1.89 ± 0.29 at 10 μM to 8.10 ± 0.60 at 1 mM (Fig. 3, strain Regular). Similar ratio values were obtained with strain Goofy (Fig. 3). These results indicate that the cleavage pathway becomes more strongly expressed than the demethylation pathway above a transition concentration of DMSP that lies between 1 and 10 μM. We propose that at this transitional concentration, the sulfur needs of the bacteria are completely met through the demethylation pathway, and excess organic sulfur at higher DMSP concentrations is released as DMS via cleavage.

### Raman microspectroscopy

The effect of sulfur satiation on cleavage pathway expression was more directly observed via single-cell Raman microspectroscopy. Measurements with deuterium-labeled DMSP revealed that bacteria that were satiated in sulfur through prior exposure to methionine (also an organic sulfur source) maintained uptake of DMSP but skewed gene expression toward the cleavage pathway (Supplementary Fig. 11). Bacterial sulfur demand has been proposed as a factor that regulates the fate of DMSP^6,7,12^. Since different elements of the DMSP molecule are harvested by the bacteria through demethylation (both carbon and sulfur) and cleavage (carbon only), our observations are consistent with the hypothesis that cells favor the cleavage pathway when they no longer require additional sulfur but continue to harvest carbon from DMSP.

### Co-incubation of phytoplankton and engineered bacteria

**Fig. 4:**
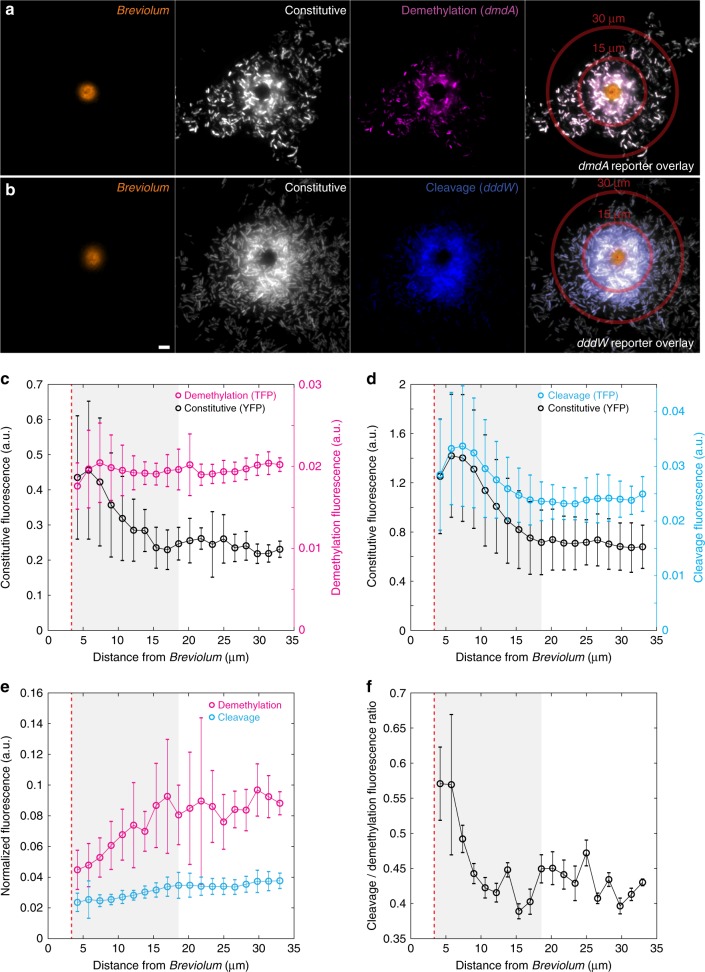
Gene expression patterns in a natural DMSP hotspot. **a**, **b** Representative images of co-incubation between DMSP-producing microalgae, *Breviolum* CCMP2459 (photosynthetic pigment, orange), and engineered bacteria, *R. pomeroyi*, constitutively expressing YFP (white) and fluorescently reporting *dmdA* (**a** demethylation, strain Regular-TFP, magenta) or *dddW* (**b** cleavage, strain Goofy-TFP, blue) expression. Fluorescence signals are false-colored. Representative concentric rings (widths, 20 pixels = 1.6 μm) that bin distances from the center (red dots) of *Breviolum* cells for fluorescence quantification are shown at 15 and 30 μm. Scale bar, 5 μm. **c**, **d** Quantification of YFP (constitutive) and TFP (*dmdA* (**c**), or *dddW* (**d**)) fluorescence at each distance (*r*) from the center of *Breviolum* cells (mean radius = 3.3 μm, red dotted line; *n* = 33). Fluorescence upregulation of YFP (**c**, **d**) and TFP (*dddW*; **d**) were detectable up to *r* = 18.6 μm (gray shading), at which modeled DMSP concentration was 35 nM (Supplementary Fig. 13). YFP of the cleavage pathway reporter (**b**, **d**) was brighter than that of the demethylation pathway reporter (**a**, **c**), probably due to differences in metabolic activity levels in each bacterial culture. Bacteria nearest to the surface of the phytoplankton (*r* = 4.2 μm, the first concentric ring) appeared dimmer than expected, possibly due to spectral interference from photosynthetic pigments. Data points and error bars represent mean ± s.d. of images (*n* = 15  *Breviolum* cells for *dmdA* (**c**); *n* = 18 for *dddW* (**d**)), calculated at each concentric ring (image analysis methods in Supplementary Note 3). **e** TFP fluorescence of each cell-containing pixel normalized by the average YFP fluorescence of cell-containing pixels within the corresponding concentric circle to remove the effect of metabolic activity differences on fluorescence intensities. Direct comparisons between demethylation and cleavage after normalization revealed that demethylation expression was higher than cleavage expression at all distances from the phytoplankton. Data points and error bars represent mean ± s.d. of normalized fluorescence of images, calculated at each concentric ring. **f** Average normalized TFP intensity (**e**) of strain Goofy (*dddW*, *n* = 18) divided by that of strain Regular (*dmdA*, *n* = 15) at each distance from the phytoplankton. Error bars represent the variance of the ratio of normalized cleavage and demethylation fluorescence signals (Supplementary Note 3).

While uptake of DMSP was detected at all concentrations tested (Supplementary Fig. 10), upregulation of DMSP degradation genes was observed only in relatively high concentrations (≥1 μM in microfluidic chip experiments; Fig. 2). DMSP is not homogeneously distributed in the water column, but often occurs as point sources of high concentration surrounding DMSP-producing organisms^24^. Bacterial exploitation of these enriched microenvironments^25^ can influence DMSP transformation rates and microbial pathway choice in the ocean. To determine DMSP degradation gene expression in the context of microscale hotspots, we exposed the *R. pomeroyi* fluorescent reporter strains to an ecologically relevant point source of DMSP: a unicellular phytoplankton. Concentration gradients of nutrients, often including DMSP, are present in the microenvironment directly surrounding phytoplankton cells (the phycosphere^24^). We co-incubated the reporter strains with the unicellular dinoflagellate *Breviolum* CCMP2459, which belongs to a family containing some of the most prolific producers of DMSP (Symbiodiniaceae; with intracellular DMSP concentrations of 36–7590 mM)^10^. Co-incubations were performed on agarose pads, which immobilized both phytoplankton and bacterial cells for ease of observation. After 24 h of co-incubation in the dark, high-magnification (100× objective) epifluorescence microscopy images of the phycosphere surrounding individual *Breviolum* cells were acquired (Fig. 4a, b and Supplementary Fig. 12). To avoid alterations to the phycosphere due to microscopy light-induced cellular stress, images were acquired at a single time point (24 h). Only teal fluorescence was quantified to represent bacterial pathway expression (due to spectral leakage in the red fluorescence channel by photosynthetic pigments, e.g., chlorophyll and carotenoids), with strain Regular reporting demethylation (*dmdA*; *n* = 15 *Breviolum* cells) and strain Goofy reporting cleavage (*dddW*; *n* = 18 *Breviolum* cells) (Fig. 4). Fluorescence intensities of bacteria were averaged across *Breviolum* cells as a function of distance from the phytoplankton cell (Supplementary Note 3).

*R. pomeroyi* gene expression patterns reflected the spatial locations of the bacteria within the phycosphere of *Breviolum* cells. According to modeled DMSP diffusion assuming a leakage rate of 11% of intracellular DMSP per day (Supplementary Note 4), the steady-state concentration at the surface of a *Breviolum* cell (radius = 3.3 ± 0.9 μm, mean ± s.d.) was 197 nM and decayed exponentially with distance, *r*, from the center of the phytoplankton cell (Supplementary Fig. 13). In line with the predicted DMSP concentration profile within the phycosphere, bacteria that were nearest to the surface of *Breviolum* cells, but far enough not to be affected by spectral interference from photosynthetic pigments (*r* = 7.4 μm), were the most metabolically active, exhibiting YFP fluorescence intensities that were on average double (1.4 ± 0.5 a.u., Goofy; 0.4 ± 0.2 a.u., Regular) those exhibited by bacteria located at *r* = 18.6 μm (0.7 ± 0.3 a.u., Goofy; 0.2 ± 0.05 a.u., Regular), beyond which YFP intensities did not change with distance (Fig. 4c, d).

The expression of the cleavage pathway (*dddW*) also increased with decreasing distance from a *Breviolum* cell. The *dddW* expression levels (3.4 ± 1.1 × 10^−2^ a.u., raw TFP signal) were highest near the surface of *Breviolum* (*r* = 7.4 μm; modeled DMSP concentration = 89 nM) (Fig. 4d) and decreased to baseline levels (2.4 ± 0.4 × 10^−2^ a.u., raw TFP signal) at *r* ≥ 18.6 μm (modeled DMSP concentration = 35 nM). These results differ from our previous microfluidic experiments where exposure to pure DMSP at <1 μM did not lead to *dddW* fluorescence upregulation (Supplementary Fig. 7). This discrepancy may be due to the presence of compounds in algal exudates that positively influence the regulation of *dddW* expression, or by the greater sensitivity of the camera setup used in the *Breviolum* experiment. In contrast, the demethylation pathway (*dmdA*) was not expressed above baseline levels at any distance from the *Breviolum* cells (Fig. 4c), but its expression, normalized by the average baseline YFP intensity (proxy for metabolic activity), was still higher than that of the cleavage pathway throughout the phycosphere (Fig. 4e). As a result, relative pathway expression was skewed towards demethylation at all distances from a *Breviolum* cell (Fig. 4f), but with decreasing distance, and thus increasing DMSP concentration, the cleavage-to-demethylation pathway ratio increased in a pattern consistent with the microfluidic observations (Fig. 3a).

These results suggest that within the phycosphere of a small phytoplankton cell slowly exuding DMSP, elevated production of DMS due to cleavage by marine bacteria occurs close to the surface of the phytoplankton cell, but most of the DMSP within the phycosphere is degraded through the demethylation pathway. However, in the scenario of a lysing phytoplankton cell that releases its intracellular DMSP at once, for example at the demise of a phytoplankton bloom, DMSP within the phycosphere can reach micro- or millimolar concentration for seconds to minutes^26^. Matching these time scales, bacterial gene expression (transcription and translation) can theoretically be upregulated within a few minutes^27^, although limitations in fluorescence signal detection prevented the observation of such early responses in the present study. Furthermore, oligotrophic DMSP degraders such as SAR11^28,29^ likely employ regulatory mechanisms that differ from copiotrophic bacteria such as *R. pomeroyi*. In general, the results from our microfluidic experiments suggest that in the vicinity of lysing phytoplankton cells (>10 μM) or in other microenvironments with similarly high DMSP levels^30^, both DMSP degradation genes increase expression, with cleavage more so than demethylation. These microscale dynamics are consistent with macroscale patterns of elevated DMS production that are observed during the decline of phytoplankton blooms^31^ and following high rates of viral-induced phytoplankton lysis^32^.

Taken together, our observations reveal that the metabolic machinery of DMSP-degrading copiotrophic bacteria may be adapted for encounters with DMSP hotspots. Baseline expression of both pathways was detected even in the absence of DMSP (Supplementary Fig. 5), possibly owing to promoter leakage of *dmdA* and *dddW*, which likely allows bacteria to be poised for the next encounter with DMSP. Upregulation of DMSP degradation genes, beyond baseline levels, was only observed at high DMSP concentrations that are characteristic of hotspots: above 1 μM for *dmdA* (Fig. 2a and Supplementary Fig. 6) and above 35 nM for *dddW* (Fig. 4b, d and Supplementary Fig. 13). Furthermore, *K*_m_ values of DMSP degradation enzymes (5.4 mM for DmdA^33^ and 4.50–8.68 mM for DddW^34^) are orders of magnitude above the mean seawater concentrations of DMSP (16.91 ± 22.17 nM^8^), further supporting the notion that bacteria are adapted to exploit sporadic encounters with DMSP hotspots^35^ as we observed by single-cell imaging.

Identifying the environmental determinants of microbial DMSP cycling is key in understanding their effects on global climate and biogeochemical cycles. Two interconnected concepts, the DMSP Availability Hypothesis^6^ and the Bacterial Switch^7^, were proposed nearly two decades ago to explain the interplay between the two DMSP degradation pathways and the factors leading to the production of DMS, but have remained largely hypothetical. The present study offers the first direct evidence that the ambient concentration of DMSP regulates the relative (i.e., cleavage-to-demethylation ratios; Figs. 3, 4), rather than mutually exclusive, expression of demethylation and cleavage pathways. We observed that elevated concentrations of DMSP (>10 μM), which are typically found in microscale hotspots, shift bacterial DMSP degradation toward cleavage and are ultimately expected to increase the bacterial production and release of DMS. Thus, we propose that the concentrations of DMSP that are most relevant for the bacterial production of DMS, and ultimately for global sulfur cycling and for the production of DMS-derived cloud condensing nuclei, may not be the levels present in bulk seawater, but instead those existing in microscale hotspots. This points to the importance of understanding the relative contribution of DMSP catabolism rates in hotspots compared to the bulk seawater, and the need to develop more realistic microscale methods to quantify the utilization and fate of this ubiquitous and important marine compound.

## Methods

### Construction of tricolor fluorescent reporter strains

Tricolor fluorescent reporters were constructed in the marine model organism *R. pomeroyi* DSS-3 (wild-type strain, a gift from Prof. M. A. Moran, University of Georgia) to visually report expression of DMSP degradation genes (*dddW* and *dmdA*). DddW was chosen due to its strong upregulation response to DMSP reported in previous transcriptomic studies^11,17^, which suggests that DddW is the primary DMSP lyase in *R. pomeroyi* DSS-3. Three fluorescent proteins were chosen for brightness, monomeric structures, and spectral separation: mTFP1 (teal)^36^; mVenus-Q69M (yellow)^37^, which is the mVenus YFP^38^ modified with a Q69M mutation to reduce environmental sensitivity; and mKate2 (far-red)^39^. To control for bias caused by the choice of color of fluorescent protein (RFP or TFP) fused to each promoter region, we constructed two *R. pomeroyi* reporter strains (Goofy and Regular) in which we interchanged the fluorescent proteins fused to *dmdA* and *dddW* promoter regions (Fig. 1a, b).

Three promoter fusion cassettes were inserted into a single vector backbone (pBBR1MCS-2, a 5.144 kb, broad-host-range, medium copy number plasmid with a kanamycin resistance cassette and origin of replication pBBR1, originally isolated from *Bordetella bronchiseptica*)^40,41^ to enable gene expression readouts from individual cells (Supplementary Fig. 14): a *dmdA* promoter reporter cassette; a *dddW* promoter reporter cassette; and a constitutively expressed *yfp* cassette (Fig. 1a, b). The 500 bp sequence upstream of the *dddW* gene and 222 bp upstream of the *dmdA* gene in the *R. pomeroyi* DSS-3 genome were determined as putative promoter regions and used to construct promoter reporter cassettes. A strong, constitutive synthetic promoter P_*A1/04/03*_ (an *E. coli lac* promoter derivative)^42,43^ controlled the expression of YFP, whose intensity was utilized as a proxy for cell viability, plasmid copy number, and metabolic activity (Supplementary Fig. 15). Transcriptional terminators (RNAI, TSAL, TR2-17, TL17, BS7, and T7TE+) and spacer regions between promoter fusion cassettes were cloned from plasmid pZS2-123^44^. As an intermediate step in the construction of the tricolor reporter strains, a derivative of pZS2-123 (with its original promoters replaced with 500 bp sequence upstream of *dmdA* or *dddW* genes), pZS2-200, was built using restriction enzymes: AvrII, XmaI, XhoI, BamHI, XmnI, and SalI (New England Biolabs).

### Construction of control reporter strains

Truncated versions of the tricolor reporters (Supplementary Fig. 1), each of which contained one of the promoter fusion cassettes, were built to test the effect of including three promoter fusion cassettes within one DNA construct (Supplementary Fig. 2). We also constructed constitutively fluorescent, single-color *R. pomeroyi* strains (Supplementary Fig. 1) to quantify spectral leakage amongst fluorescent protein colors, and to calculate the spectral leakage correction matrix, *B* (Supplementary Note 1 and Supplementary Fig. 16).

### DNA assembly

All *R. pomeroyi* strains and plasmids engineered in this study are listed in Supplementary Table 1 and Supplementary Fig. 1. For assembly of the promoter fusion cassettes into the pBBR1MCS-2 vector, DNA fragments containing overlapping regions (~30 bp) were amplified with 25 cycles of polymerase chain reaction (PCR) using KAPA HiFi HotStart ReadyMix (Kapa Biosystems) with primers listed in Supplementary Table 2. Putative promoter regions of *dmdA* and *dddW* were amplified from *R*. *pomeroyi* DSS-3 genomic DNA isolated with DNeasy Blood & Tissue Kits (Qiagen). Plasmids that were used as PCR templates (Supplementary Table 3) were isolated using the QIAprep Spin Miniprep Kit (Qiagen). Primers for vector backbone amplification were designed to eliminate the multiple cloning site (MCS) within the *lacZα* gene in the pBBR1MCS-2 vector to prevent fusion with the β-galactosidase *α*-peptide. Two extra stop codons (TAATAA) were added to each fluorescent protein gene sequence through primer design.

DNA fragments were assembled using NEBuilder HiFi DNA Assembly Master Mix (New England Biolabs). Assembled plasmids were transformed into electrocompetent *E. coli* (NEB 10-beta; New England Biolabs) through electroporation (Gene Pulser Xcell, Bio-Rad), positive colonies were picked on X-Gal/IPTG Luria Broth (LB) plates, and correct assembly of DNA fragments was confirmed through sequencing of purified plasmids using diagnostic primers listed in Supplementary Table 2.

### Transformation of *R. pomeroyi* through conjugation

Reporter plasmids were transformed into *R. pomeroyi* DSS-3 through a triparental conjugation method, which was found to be ideal due to the large sizes of our reporter plasmids (up to 7.974 kb). Overnight liquid culture of wild-type *R. pomeroyi* was prepared in half-strength YTSS (1/2 YTSS) containing (500 ml^−1^) 2 g yeast extract (BD Biosciences), 1.25 g tryptone (BD Biosciences), 10 g sea salts (Sigma-Aldrich). In addition, overnight liquid cultures of helper *E. coli* containing the pRK600 plasmid^45^ (15 μg ml^−1^ chloramphenicol) and donor *E. coli* containing a constructed reporter plasmid (50 μg ml^−1^ kanamycin) were prepared in LB. *E. coli* cultures were washed twice in 1/2 YTSS medium to eliminate antibiotics. The following mixture was concentrated and resuspended in a final volume of 100 μl 1/2 YTSS medium: 2 ml overnight culture of *R. pomeroyi*, 200 μl of washed overnight culture of helper *E. coli*, and 200 μl of washed overnight culture of donor *E. coli*. 50 μl of this bacterial mixture was spotted on a 1/2 YTSS plate, and incubated overnight at 30 °C to allow mating to occur. Selection for plasmid-containing *R. pomeroyi* was achieved by re-streaking onto a 1/2 YTSS plate amended with kanamycin (50 μg ml^–1^) and potassium tellurite (50 μg ml^–1^; Fluorochem). Like many marine microorganisms^46,47^, *R. pomeroyi* was found in this study to be resistant to potassium tellurite, while *E. coli* is known to be sensitive to the oxide mineral. Successfully transformed *R. pomeroyi* were confirmed through colony PCR and sequencing.

### Bacterial culture preparation for experiments

A frozen glycerol stock of each *R. pomeroyi* reporter strain was streaked onto a half-strength YTSS (1/2 YTSS) culture plate containing (500 ml^−1^) 7.5 g agar (Bacto Agar, BD Biosciences), amended with 50 μg ml^−1^ kanamycin sulfate (Sigma-Aldrich), and incubated at 30 °C for 48–72 h. A single colony was picked from the plate to inoculate 1 ml of 1/2 YTSS liquid medium amended with 25 μg ml^−1^ kanamycin for overnight culture (~19 h) in the dark at 30 °C on an orbital shaker (200 rpm). When overnight cultures reached visible turbidity, subcultures were prepared by washing (6300 × *g*, 3 min) and diluting (1/75 vol/vol) overnight culture cells to a final volume of 1.5 ml in marine basal medium (MBM) consisting of 0.07 M Tris HCl (pH 7.5), 0.24 mM K_2_HPO_4_, 13.4 mM NH_4_Cl, 0.073 mM FeEDTA, 2% (wt/vol) sea salts, ﻿and 0.1% (vol/vol) vitamin solution^48^. d-glucose (10 mM) was provided as a sole amended carbon source in MBM, and kanamycin (25 μg ml^−1^) was added for plasmid retention. Initial OD_700_ of 10 mM glucose MBM subcultures ranged from 0.04 to 0.05. After a 4-h incubation in similar conditions as overnight cultures, OD_700_ decreased to 0.03 to 0.04, probably due to a decrease in cell size as cells transitioned from rich medium (elongated cells) to MBM (shortened cells).

To prepare for the low DMSP concentration (≤1 μM) experiment (Supplementary Fig. 7), several colonies of strain Regular cells on an agar plate were resuspended directly into MBM amended with 1 mM succinate and 50 μg ml^–1^ kanamycin, and grown overnight at 30 °C in the light. The overnight culture was diluted (1/50 vol/vol) into fresh 1 mM succinate MBM with kanamycin (50 μg ml^–1^) and incubated for an additional 4 h. Similar to other time-lapse experiments, OD_700_ was 0.3 before initiation of incubation with DMSP.

### Carbon sources test

To assess the validity of engineered reporter strains, as well as to identify an appropriate negative control carbon source, *R. pomeroyi* reporter strains Regular and Goofy were incubated with a range of carbon sources, and their fluorescence response and growth were measured (Supplementary Fig. 3). Carbon sources chosen for this experiment were utilized in previous studies to cultivate *R. pomeroyi*. MBM solutions amended with 10 mM of the following carbon sources were prepared: DMSP (Tokyo Chemical Industry), sodium succinate dibasic hexahydrate (Sigma-Aldrich), sodium propionate (Sigma-Aldrich), sodium acetate (Sigma-Aldrich), sodium acrylate (Sigma-Aldrich), or d-glucose (Sigma-Aldrich). MBM solutions were filter sterilized (0.2 μm) after dissolution of carbon sources. The 5% 1/2 YTSS was prepared with a dilution (1/20 vol/vol) of the rich medium in non-carbon amended MBM.

Several colonies of *R. pomeroyi* reporter strains on agar plates were washed and resuspended in non-carbon amended MBM. Resuspended cells (2 μl) were seeded into 0.75 ml of each carbon source MBM solution amended with 25 μl ml^−1^ kanamycin. Incubations were performed in 2-ml microcentrifuge tubes (Eppendorf) in the dark at 30 °C with 200 rpm orbital shaking for 18.5 h before microscopy imaging.

Glucose was chosen as the most suitable negative control for the following reasons: it elicited no non-specific DMSP gene transcription response (Supplementary Fig. 3); its molecular weight is similar to DMSP; and the metabolic pathways of glucose and organosulfur compounds are distinct. The low DMSP concentration (≤1 μM) experiment (Supplementary Fig. 7) was the only instance in which succinate was used as the negative control, as it had been utilized in previous studies^21,49^. While succinate was also suitable as negative control (Supplementary Fig. 3), it produced slightly higher non-specific fluorescence response than glucose; thus, glucose, unless otherwise noted, was utilized as the negative control for all other experiments.

### Growth curves

Growth curves of *R. pomeroyi* strains Regular and Goofy in different carbon sources (Supplementary Fig. 3) were measured in a flat-bottom 96-well plate (Thermo Fisher Scientific), containing 200 μl of carbon source-amended MBM per well in the absence of antibiotics. Each carbon source was prepared in triplicates, with corresponding blank wells in duplicates. Each well was inoculated with 2 μl of bacteria, grown overnight in 1/2 YTSS amended with 25 μg ml^−1^ kanamycin, and washed and resuspended without dilution in non-carbon amended MBM. Optical density was measured at 700 nm (OD_700_) to avoid spectral interference from fluorescence. The plate was incubated at 25 °C, and OD_700_ was measured every 1 h (3 min of fast orbital shaking before each time point) for 56 h using a Synergy HTX Multi-Mode Microplate Reader (BioTek Instruments).

### Microfluidic device fabrication

The microfluidic device containing nine parallel observation chambers (Fig. 1c) was fabricated using soft lithography^50^. A mold for the observational chamber geometry was fabricated with SU8 on a silicon wafer. The microfluidic device was then created by casting polydimethylsiloxane (PDMS) (SYLGARD 184 Silicone Elastomer Kit; Dow Corning) onto the mold. The cured PDMS was then removed from the mold, perforated with inlet and outlet holes with a biopsy punch (1.5 mm diameter), and permanently fixed to a glass coverslip (60 mm × 24 mm; 0.17 ± 0.005 mm precision thickness; Carl Roth) by plasma bonding. Depth of each observation chamber was ~60 μm.

### Microscopy

All experiments were performed using an inverted epifluorescence TE2000 microscope (Nikon) controlled through Nikon Elements software (unless otherwise specified). A Spectra X LED light source (Lumencore) provided single wavelength excitation illumination for fluorescence imaging (100% LED power unless otherwise indicated). The Perfect Focus System (Nikon) was engaged to maintain focus in time-lapse experiments. Three filter cubes (Chroma) were used for fluorescence imaging: a custom filter cube optimized for mKate2 RFP (ET580/25x excitation filter, T600lpxr band-pass filter, and ET645/75m emission filter), Chroma 49003 for YFP, and Chroma 49013 for TFP. Unless otherwise indicated, the excitation filter of each cube was removed for imaging to maximize fluorescence signal captured. At each field of view, phase contrast and fluorescence images were captured sequentially in the following order: phase contrast, red fluorescence channel (575 nm excitation), yellow fluorescence channel (508 nm excitation), and teal fluorescence channel (440 nm excitation). Bacteria were introduced into microfluidic devices, allowed to settle for 20–30 min, and imaged at the plane of the glass coverslip surface.

Images were acquired with an electron multiplying CCD (EMCCD) camera (iXon_3_ 885; Andor Technology) (1004 × 1002 pixels; 8 μm pixel size) for the following experiments: time-lapse DMSP experiments in microfluidic chips; large-volume DMSP concentration measurement experiment (Supplementary Fig. 10); carbon sources test (Supplementary Fig. 3); DMSP uptake experiment (Supplementary Fig. 11); and phytoplankton co-incubation (Fig. 4 and Supplementary Fig. 12). For time-lapse DMSP experiments in microfluidic chips and the large-volume DMSP concentration measurement experiment, a 40× objective (CFI S Plan Fluor ELWD ADM 40×, correction collar adjusted to 0.17; Nikon) was used with electron multiplier gain at 3×, and the following exposure times: phase contrast (20 ms, 5% white LED power), red (100 ms), yellow (100 ms), and teal (200 ms). Imaging conditions for the carbon sources test were identical, except exposure time for teal fluorescence imaging was 100 ms. Time-lapse imaging for the DMSP uptake experiment was done in parallel with, but on a different microscope from, Raman microspectroscopy measurements (see below). Only phase contrast and teal fluorescence were acquired, with microscopy setup as described above. For phytoplankton co-incubation imaging, an oil-immersion 100× objective (CFI Plan Apo Lambda DM 100× Oil; Nikon) was used, without electron multiplier gain, with the following exposure times (total 240 ms per image) and LED powers: phase contrast, 60 ms, 10%; red, 100 ms, 100%; yellow, 40 ms, 50%; and teal, 40 ms, 100%.

Finally, images for the low DMSP concentration (≤1 μM) experiment (Supplementary Fig. 7) were acquired with an sCMOS camera (Zyla 4.2; Andor Technology) (2048 × 2048 pixels; 6.5 μm pixel size). A 40× objective (described above) was used, with the following camera exposure times: phase contrast, 9.8 ms, 10% white LED power; and all fluorescence channels, 200 ms.

### Image analysis

Analysis of fluorescence images was performed in MATLAB (MathWorks) using an automated image segmentation and fluorescence quantification software developed in-house. Detailed descriptions of image processing and analysis methodologies for microfluidic and agarose pad co-incubation experiments are provided in Supplementary Notes 1 and 3, respectively. Briefly, cells were segmented by pixel intensity thresholding in phase contrast images. Background subtraction and spectral leakage correction were performed to enable accurate quantification of cellular fluorescence. Thresholding on YFP fluorescence intensity (proxy for metabolic activity) was applied to only include viable cells for further analyses. Finally, fluorescence signals in red and teal channels of each cell were normalized by the mean YFP signal at each time point of each experimental condition.

### DMSP pathway expression time-lapse experiment

Each replicate experiment represents a biological replicate performed on a single microfluidic device containing nine observation chambers (Fig. 1c). For each replicate experiment, one of the two *R. pomeroyi* reporter strains (Regular or Goofy) was prepared for experimentation as described above. At the end of subculture incubation, cells were washed and concentrated by 4.5× in non-carbon amended MBM amended with kanamycin (10 μg ml^−1^), and distributed into nine separate microcentrifuge tubes (Eppendorf) representing each treatment condition. For each tested concentration of glucose or DMSP, a 10× concentrated stock solution was prepared in non-carbon amended MBM amended with kanamycin (10 μg ml^–1^). To initiate incubation, 10× stock solutions were diluted to 1× final concentration in the cell-containing MBM, resulting in a 4.05× cumulative concentration of subcultured cells. Each observation chamber was populated with 12.5 μl of treated cells. Inlet and outlet holes of observation chambers were sealed with clear tape to minimize evaporation. Since PDMS is a gas-permeable material, oxygen is not expected to be limited in our experimental setup. Cells in observation chambers were allowed to settle onto the glass coverslip surface with gravity for 20–30 min before initiation of image capture. Phase contrast and fluorescence images were captured at seven positions, determined manually before start of imaging, per observation chamber every 45 min for ~24 h. Replicate experiment 3 of strain Regular (Supplementary Figs. 4, 6, 15) contained 6 imaging positions (instead of 7) per observation chamber. All fluorescence kinetic experiments, except the low DMSP concentration (≤1 μM) experiment, were conducted at room temperature (21 °C) and in the dark. In the low DMSP concentration (≤1 μM) experiment only (Supplementary Fig. 7), 1 mM succinate was used as negative control, all experimental conditions contained 50 μg ml^−1^ kanamycin, and fluorescence was monitored by microscopy with image acquisition every 30 min for 7.4 h with a cage incubator set to 30 °C.

### Cultivation of phytoplankton

The dinoflagellate *Breviolum* (strain CCMP2459, formally within genus *Symbiodinium*^51^) was chosen for its prolific production of DMSP. *Breviolum* cells were grown in sterile plastic culture flasks (Nunclon EasyFlasks, 25 cm^3^ volume; Thermo Fisher Scientific) under a diel light cycle (14 h light:10 h dark, (100 μmol m^−2^ s^−1^)) in 30 ml *f*/2 medium at 22 °C. Cells at 22 days post-inoculation (a 1:100 dilution into fresh medium) were harvested for experimentation at 14:00 in the afternoon. Cellular concentration was determined by counting in a microfluidic observation chamber (21 cells μl^–1^).

### Phytoplankton–bacteria co-incubation experiment

Co-incubations between *Breviolum* cells and *R. pomeroyi* reporter strains Regular or Goofy were performed on agarose pads, which immobilized the algal cells and allowed them to establish their phycospheres (i.e., the immediate regions surrounding unicellular algae cells) through steady exudation. For agarose pad preparation, low melting temperature agarose (Promega) was combined with 1/2 YTSS medium at 15 mg ml^−1^, and gently dissolved in a microwave. After partial cooling, kanamycin was added at 25 μg ml^−1^ final concentration. Rubber gaskets (0.5 mm thickness) were manually cut into square frames (~2 cm × 2 cm inner square area) and placed on glass coverslips (22 mm × 50 mm; VWR). The inner square areas of rubber gaskets were filled with ~500 μl melted agarose–kanamycin mixture. Agarose pads were allowed to cool and solidify for 1.5 h before seeding with *R. pomeroyi* strains.

Bacteria were first seeded onto agarose pads and allowed to grow for 24 h without phytoplankton. Overnight cultures of *R. pomeroyi* strains grown in 1/2 YTSS were washed and concentrated threefold in non-carbon amended MBM amended with 10 μg ml^–1^ kanamycin. Ten microlitre of this concentrated cell mixture was spotted onto the center of each agarose pad, and loosely covered with a plastic lid (without contacting the agarose) to minimize evaporation. *R. pomeroyi* strains were allowed to grow in patches of monolayer cells on the agarose pads for 24 h at 30 °C in the light before *Breviolum* cells were added to initiate co-incubation.

In preparation for co-incubation with bacteria, *Breviolum* cells were washed and concentrated 20-fold in fresh *f*/2 medium amended with 10 μg ml^−1^ kanamycin. Prepared *Breviolum* cells (10 μl) were spotted onto the middle of each agarose pad containing monolayer growth of *R. pomeroyi*. Co-incubation agarose pads were incubated at room temperature (21 °C) in the dark, loosely covered with a plastic lid without contacting the agarose, for 24 h before imaging. Prior to imaging, a glass coverslip (60 mm × 24 mm; 0.17 ± 0.005 mm precision thickness; Carl Roth) was placed onto the agarose pads carefully to avoid agitation of established phycospheres, and flipped onto the oil immersion objective for imaging. Only one time point (24 h) was taken for microscopy, to avoid microscopy light-induced cell stress that could alter the phycosphere profile.

To calculate the spectral leakage correction matrix (*B*_agarose_; Supplementary Note 1), different colors of single-color constitutive control strains of *R. pomeroyi* (Supplementary Fig. 1) were grown on separate agarose pads for 24 h without phytoplankton, and imaged as described above.

### Large-volume DMSP concentration measurement experiment

To estimate the DMSP concentration evolution in microfluidic observation chambers over time, a large-volume (8 ml) experiment was performed to allow sampling for DMSP concentration measurements (Supplementary Fig. 10). The experiment, while larger in volume by ~800-times compared to microfluidic experiments, preserved cell-to-volume ratio at all steps in the protocol. Three representative initial concentrations of DMSP were chosen, each of which was incubated in triplicates: 1 μM, 75 μM, and 1 mM.

*R. pomeroyi* (strain Regular) was grown and prepared as described above, with modifications as described below. Three biological replicates (i.e., three different colonies as inocula) of overnight cultures were prepared in 2.5 ml of 1/2 YTSS rich medium per replicate. Overnight culture cells were washed and concentrated tenfold in non-carbon amended MBM. For each biological replicate, 153 μl of concentrated overnight culture was used to inoculate subculture flasks containing 115 ml of 10 mM glucose MBM amended with 25 μg ml^–1^ kanamycin. This led to a cumulative dilution factor of 1/75 (vol/vol) of overnight culture for subculture preparation, consistent with microfluidic experiments. Subcultures were incubated in the dark at 30 °C on an orbital shaker (200 rpm) for 4 h, at the end of which OD_700_ was measured to be 0.02–0.04. A volume of 110 ml of subcultured cells per biological replicate was washed and concentrated fivefold in non-carbon amended MBM and 7.2 ml of this concentrated cells was allocated into each treatment flask of each biological replicate (150-ml glass Erlenmeyer flasks). The addition of 800 μL concentrated DMSP solution stocks (10× concentration, i.e., 10 μM, 750 μM, and 10 mM) and kanamycin (final concentration = 10 μg ml^−1^) marked the initiation of incubation, with a starting volume of 8 ml. Final cumulative concentration from subcultured cells was 4.5× (nearly consistent with microfluidic experiments). One replicate of blank control flasks (i.e., without cells) representing each DMSP concentration condition was also prepared. All experimental flasks were sealed and incubated in the dark at room temperature (21.4–22.8 °C) and in the absence of agitation.

At each sampling time point, incubation flasks were swirled to resuspend sunken cells. From each flask, a 1.5-ml sample was taken for DMSP concentration measurement, and an additional 10-μl sample was placed in a microfluidic observation chamber for microscopy observation (imaged as described above). The first time point (0 h) was taken from the blank control flasks, for measurement of initial DMSP concentration. Subsequent time points, at which samples were taken from blank control flasks as well as from all replicate experimental conditions, were approximately 2, 8, and 24 h after the start of incubation with DMSP.

Each sample was immediately centrifuged at 2500 × *g* for 3 min to remove cells from solution. One millilitre of the supernatant was placed in an acid-washed 5-ml glass scintillation vial containing 3 ml methanol (>99.9%, HPLC gradient grade; VWR International). Sample vials were sealed and stored in the dark at 4 °C until DMSP concentration measurement using ultra-high-pressure liquid chromatography/high-resolution mass spectrometry (UHPLC/HRMS).

### Chromatography/high-resolution mass spectrometry

To prepare samples for DMSP concentration measurements, 50 μl of each sample (processed and stored as described above) was diluted with 100 μl of a mixture of acetonitrile and water (9:1 v/v), centrifuged (4500 × *g*, 5 min), and the supernatant was used for UHPLC/HRMS measurements. All UHPLC/HRMS results were obtained on a Dionex Ultimate 3000 system (Thermo Scientific) coupled to a Q Exactive Plus Orbitrap mass spectrometer (Thermo Scientific).

UHPLC/HRMS quantification followed a previously reported protocol^49^: the eluent consisted of high-purity water with 2% acetonitrile and 0.1% formic acid (solvent A) and 90% acetonitrile with 10% 5 mmol l^−1^ aqueous ammonium acetate (solvent B). The flow rate was set to 0.60 ml min^−1^. A linear gradient was used for separation with 100% solvent B (1 min), 20% B (6.5 min), 100% B (7.1 min), and 100% B (10 min). The LC separation column (SeQuant ZIC-HILIC column (5 mm, 2.1 × 150 mm) equipped with a SeQuant ZIC-HILIC guard column (5 mm, 2.1 × 20 mm)) was kept at 25 °C. Electrospray ionization was performed in positive mode ionization, recording the mass range from 75 to 200 *m*/*z*, with the following parameters: capillary temperature 380 °C; spray voltage 3000 V; sheath gas flow 60 arbitrary units; and aux gas flow 20 arbitrary units. The injection volume was 2 μl.

Calibration curves for DMSP were recorded in triplicate using synthetic standards prepared as described in a previous study^49^. Calibration curve for DMSP: area [DMSP] = 470,540*c* [DMSP in nM] with *r* = 0.9999. Data analyses were performed using the software Thermo Xcalibur version 3.0.63.

### Pre-exposure to sulfur experiment with Raman microspectroscopy

Raman microspectroscopy was utilized to infer uptake of DMSP at the single-cell level by measuring the deuterium-labeling status of cells incubated with deuterated DMSP ([^2^H_6_]-DMSP), in which the two CH_3_ groups of the DMSP molecule were labeled with deuterium in a protocol previously reported^52^ (Supplementary Fig. 11). Three incubation conditions were tested to probe the effect of sulfur satiation (due to pre-exposure of *R. pomeroyi* to 10 mM methionine) on DMSP uptake and cleavage pathway expression: [^2^H_6_]-DMSP without pre-exposure to methionine; [^2^H_6_]-DMSP with pre-exposure to methionine; and non-labeled DMSP without pre-exposure to methionine (negative control for Raman microspectroscopy signal).

The P_*dddW*_::mTFP1 single-color *R. pomeroyi* reporter strain (cleavage pathway promoter-fusion with TFP) was used to avoid spectral interference with Raman microspectroscopy measurements (see below). An overnight culture in rich medium was prepared as described above. Two subculture conditions, with or without 10 mM l-methionine (Sigma-Aldrich), were prepared in 10 mM glucose MBM amended with 25 μg ml^–1^ kanamycin, and incubated for 4 h as described above. Subcultured cells were washed, concentrated threefold, and resuspended in the appropriate solution for incubation: 1 mM [^2^H_6_]-DMSP MBM or 1 mM unlabeled DMSP MBM. All incubation conditions contained final concentrations of 1% methanol (solvent in which [^2^H_6_]-DMSP was dissolved) and 10 μg ml^–1^ kanamycin. Treated cells were incubated at room temperature (22.8 °C) in the dark for 5.5 h before imaging and Raman microspectroscopy measurements.

We utilized a commercial confocal Raman microspectroscope (LabRAM HR Evolution; HORIBA Scientific) based on an inverted microscope (Eclipse Ti; Nikon) with two cameras: an sCMOS camera (ORCA-Flash 4.0; Hamamatsu Photonics; field of view of 221.867 μm × 221.867 μm; camera #1) for fluorescence and brightfield measurements with high sensitivity; and a CMOS camera (UI-3580LE; IDS Imaging Development Systems GmbH; camera #2) for positioning the Raman laser (532-nm neodymium-doped yttrium garnet—Nd:YAG) onto each cell. After incubation of cells with deuterated or non-deuterated DMSP as described above, a 4 μl-droplet of cells was placed on a CaF_2_ coverslip (25 mm × 15 mm × 0.2 mm; Crystran), which was chosen to avoid background Raman signal noise^53^. A thin liquid column containing cells was achieved by separating the bottom (CaF_2_) and top (glass, 18 mm × 18 mm, no. 1 thickness) coverslips with 0.17 mm-thick glass coverslips placed along two opposing edges of the setup. As the Raman measurements took place at the surface of the CaF_2_ coverslip (i.e., 170 μm away from glass), this arrangement generated sufficient thickness of liquid sample in the *z*-direction to prevent interference from the glass material of the top coverslip^54^. The coverslip setup was secured onto the microscope stage with the CaF_2_ surface interfacing with the 60× water-immersion objective (Plan Apo IR 60XC 1.27 WI; Nikon), and was left undisturbed for 10 min to allow cells to settle to the bottom surface with gravity.

To measure the expression of the cleavage pathway, a fluorescence image in the teal channel (filter cube Chroma 49013 with excitation (445/30 nm) and emission (500/40 nm) filters installed to minimize interference with the Raman measurements performed with the 532 nm laser) was first acquired with camera #1 (50 ms exposure). A matching brightfield image (2 ms exposure, white LED light source) was also captured to visualize all cells. After fluorescence and brightfield image capture, the system was shifted to the Raman configuration (i.e., Raman laser and camera #2).

Single-cell Raman measurements were performed (1.5-s exposure time; 150-μm pinhole size) by manually moving the *xy*-stage to align the Raman laser (532 nm, 400 mW power), as well as to focus it (confirmed via inspection with camera #2), onto each cell. For each experimental condition sample, Raman measurements of as many cells as possible were taken within 30 min (*n* = 32–52 cells per treatment condition). Each cell’s Raman measurement and fluorescence signal were retrospectively matched using the brightfield image as the reference.

To determine the presence of DMSP uptake by cells, the DMSP uptake index (*P*_DMSP_) was computed for each cell

$$P_{\rm{DMSP}} = \frac{{I_{2040 - 2300}}}{{I_{2400 - 2450}}},$$

where *I*_2040–2300_ (numerator) and *I*_2400–2450_ (denominator) represent the integrated intensities in the Raman spectrum regions between the wavenumbers 2040 and 2300 cm^–1^ (the C–D peak whose intensity is affected by deuterium^55^) and between 2400 and 2450 cm^−1^ (reference region where background intensity was low), respectively.

### Reporting summary

Further information on research design is available in the Nature Research Reporting Summary linked to this article.

## Supplementary information

Supplementary Information

Supplementary Table 2

Reporting Summary

Source Data 1

## Data Availability

The data that support the findings of this study are available from the corresponding authors on request (total data size approximately 1 TB). The source data underlying Figs. 2, 3, and 4c–f are provided as a Source Data file.

## Code availability

All computer code (in MATLAB) developed for this study is available from the corresponding authors on request.
