# Supplementary material for: Single-cell bacterial transcription measurements reveal the importance of dimethylsulfoniopropionate (DMSP) hotspots in ocean sulfur cycling
Source: Nat Commun. 2020 Apr 23;11:1942. doi: 10.1038/s41467-020-15693-z (PMC7181598; doi:10.1038/s41467-020-15693-z)
Supplement: Supplementary file 1 — Supplementary Information [file 41467_2020_15693_MOESM1_ESM.pdf]

Supplementary Information for

Single-cell bacterial transcription measurements reveal the importance of dimethylsulfoniopropionate (DMSP) hotspots in ocean sulfur cycling

by

Gao *et al.*

Supplementary Figures

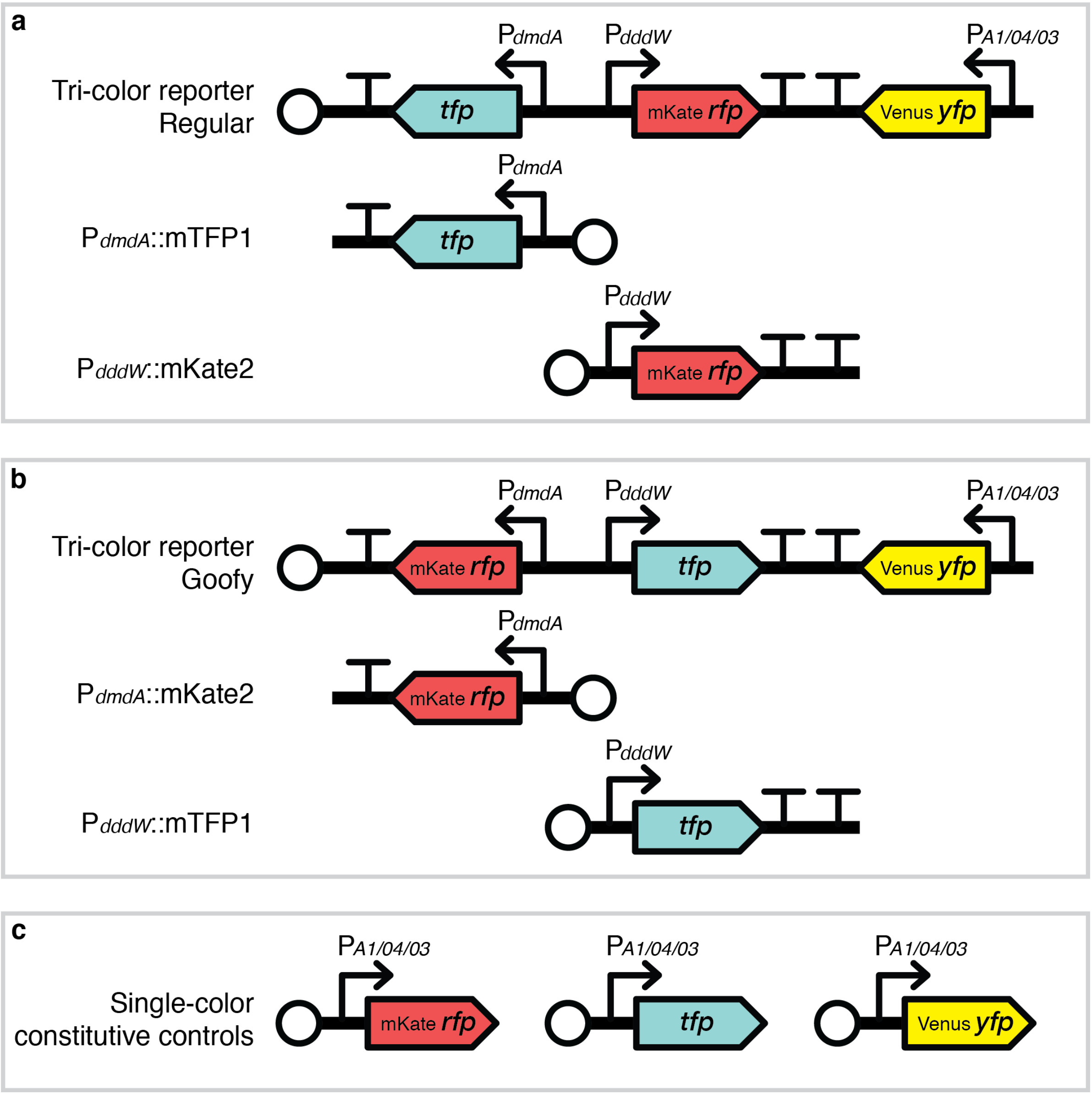

Supplementary Fig. 1 All engineered fluorescent *R. pomeroyi* DSS-3 strains used in this study. Two tricolor reporter strains, Regular (**a**) and Goofy (**b**), with interchanged colors of fluorescent proteins fused to *dmdA* and *dddW* promoter regions were built to control for spectral bias during fluorescence intensity quantification. Four single-color reporter strains, representing truncated versions Regular and Goofy, were built to verify that incorporating three promoter fusion cassettes within one DNA construct did not affect our conclusions (Supplementary Fig. 2): P_{dmdA}::mTFP1 and P_{dddW}::mKate2 (truncated Regular, **a**) and P_{dmdA}::mKate2 and P_{dddW}::mTFP1 (truncated Goofy, **b**). Finally, a spectral leakage correction matrix, *B* (Supplementary Note 1), was calculated using images of constitutively fluorescent single-color control strains (**c**). Vector backbone: pBBR1MCS-2 with origin of replication pBBR1 (open circles).

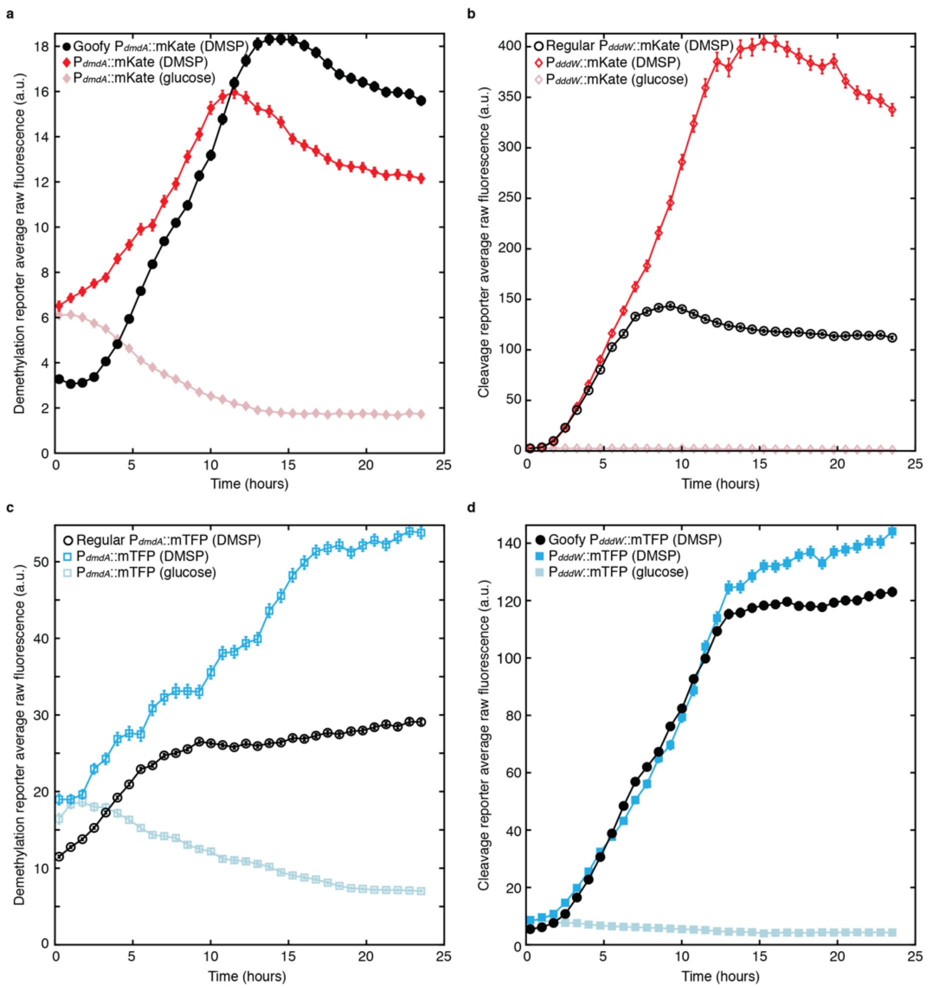

Supplementary Fig. 2 Tricolor and truncated single-color reporter strains yield similar fluorescence responses in the presence of DMSP. To confirm that the incorporation of three promoter fusion cassettes within one DNA construct (*i.e.*, in tricolor reporter strains Regular and Goofy) did not affect our overall conclusions, four single-color reporter strains, representing truncated versions of tricolor reporter strains Regular and Goofy, were incubated with DMSP or glucose (1 mM), and their fluorescence signals were recorded: P_{dmdA}::mKate2 (**a**); P_{dddW}::mKate2 (**b**); P_{dmdA}::mTFP1 (**c**); and P_{dddW}::mTFP1 (**d**). Tricolor reporters Goofy (**a,d**) and Regular (**b,c**) were also incubated with DMSP in the same experiment for comparison. Image segmentation and background subtraction were performed as described in Methods. For tricolor reporters only, spectral leakage correction and YFP thresholding (at 25 a.u.) were performed, but normalization by constitutive YFP was not (*i.e.*, raw fluorescence). While absolute magnitudes and slopes of fluorescence responses were not identical between tricolor and truncated single-color reporter strains (probably due to slight differences in physiological states in different bacterial cultures), and the choice of fluorescent protein led to slight differences in the temporal evolution of fluorescence signals, the general patterns of fluorescence kinetics were preserved (*i.e.*, initial lag, followed by linear increase that flattens after ~8–17 h; further discussion in Supplementary Note 2). These conserved behaviors validate the tricolor reporters' design and accuracy of their fluorescence signals in reporting *dmdA* and *dddW* expression dynamics and relative expression levels. Data points and error bars represent mean ± s.e.m. of raw fluorescence intensities of cells.

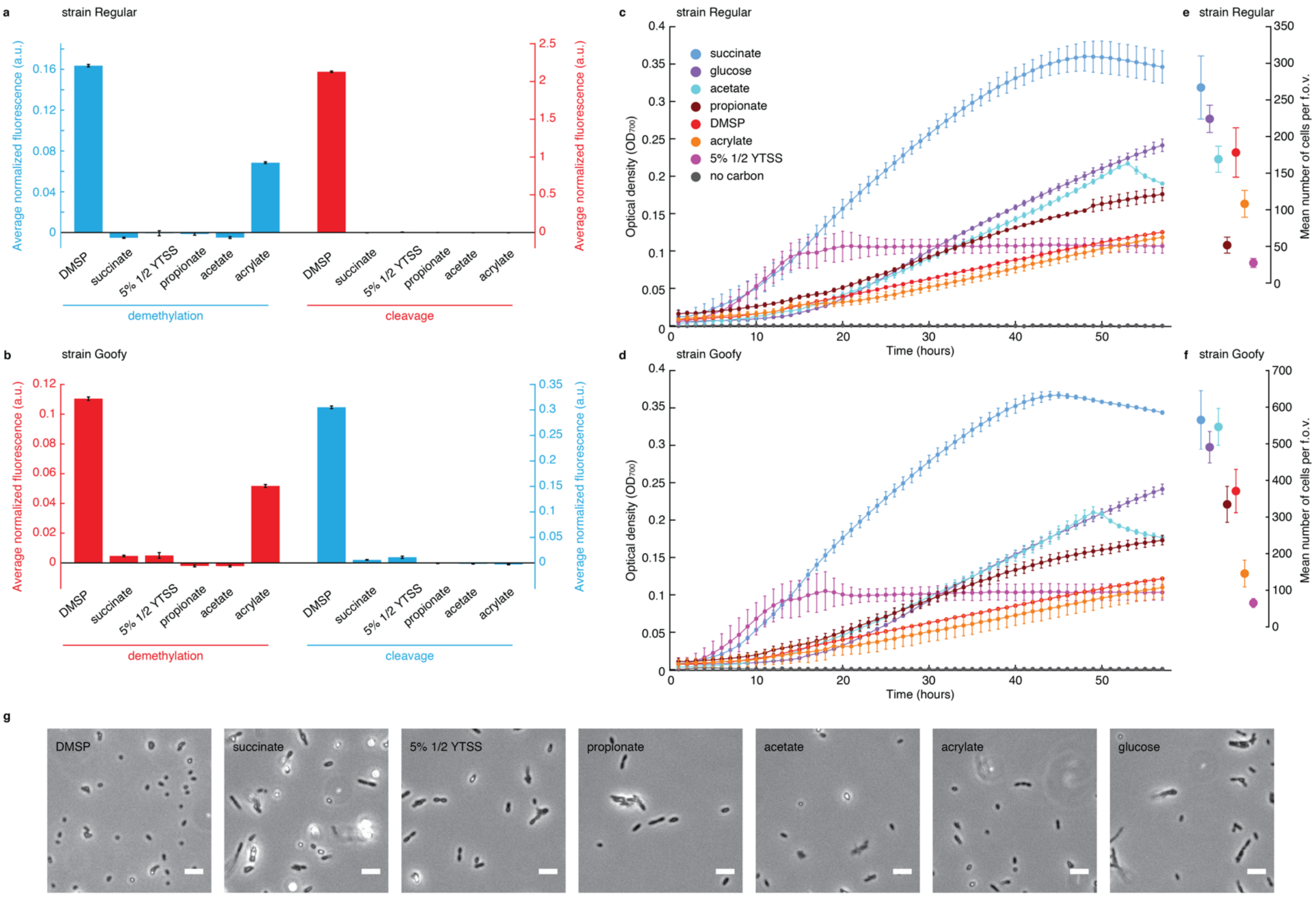

Supplementary Fig. 3 Fluorescence response and growth of engineered *R. pomeroyi* reporters in diverse carbon sources. **a** and **b** Mean fluorescence intensities of strains Regular (**a**) and Goofy (**b**) after incubation with DMSP; succinate; 5% rich media (1/2 YTSS); propionate; acetate; acrylate; or glucose provided as sole amended carbon sources for 18.5 h in the presence of antibiotic pressure (25 μM kanamycin; 30 °C; continuously shaken). All carbon sources were provided at 10 mM concentration except rich media (1/2 YTSS). YFP threshold of 10 a.u. was applied, and each cell's teal and red fluorescence were normalized by its own yellow fluorescence. After spectral leakage correction and background fluorescence subtraction, mean fluorescence values of glucose (used as negative controls in experiments) were subtracted from each cell's signals in corresponding color channels before calculation of mean; thus, some mean fluorescence values were negative. Bars and error bars represent mean ± s.e.m. of fluorescence signals of cells. **c** and **d** Growth curves of *R. pomeroyi* reporter strains incubated with different organic compounds as sole amended carbon sources in the absence of antibiotic pressure (25 °C). Data points and error bars represent mean ± s.d. of blank-subtracted triplicate wells. **e** and **f** Average number of YFP-positive cells per field of view (f.o.v.; 200 μm × 200 μm) in images acquired for fluorescence quantification (**a**,**b**). Example phase microscopy images are shown in **g** (strain Regular; scale bars, 5 μm). Relative growth yields according to optical density (**c**,**d**; OD_{700}) were consistent with cell counts (**e**,**f**) except in DMSP, due to their smaller cell size compared to bacteria grown in other carbon sources. Data points and error bars represent mean ± s.d. of cell number in a field of view.

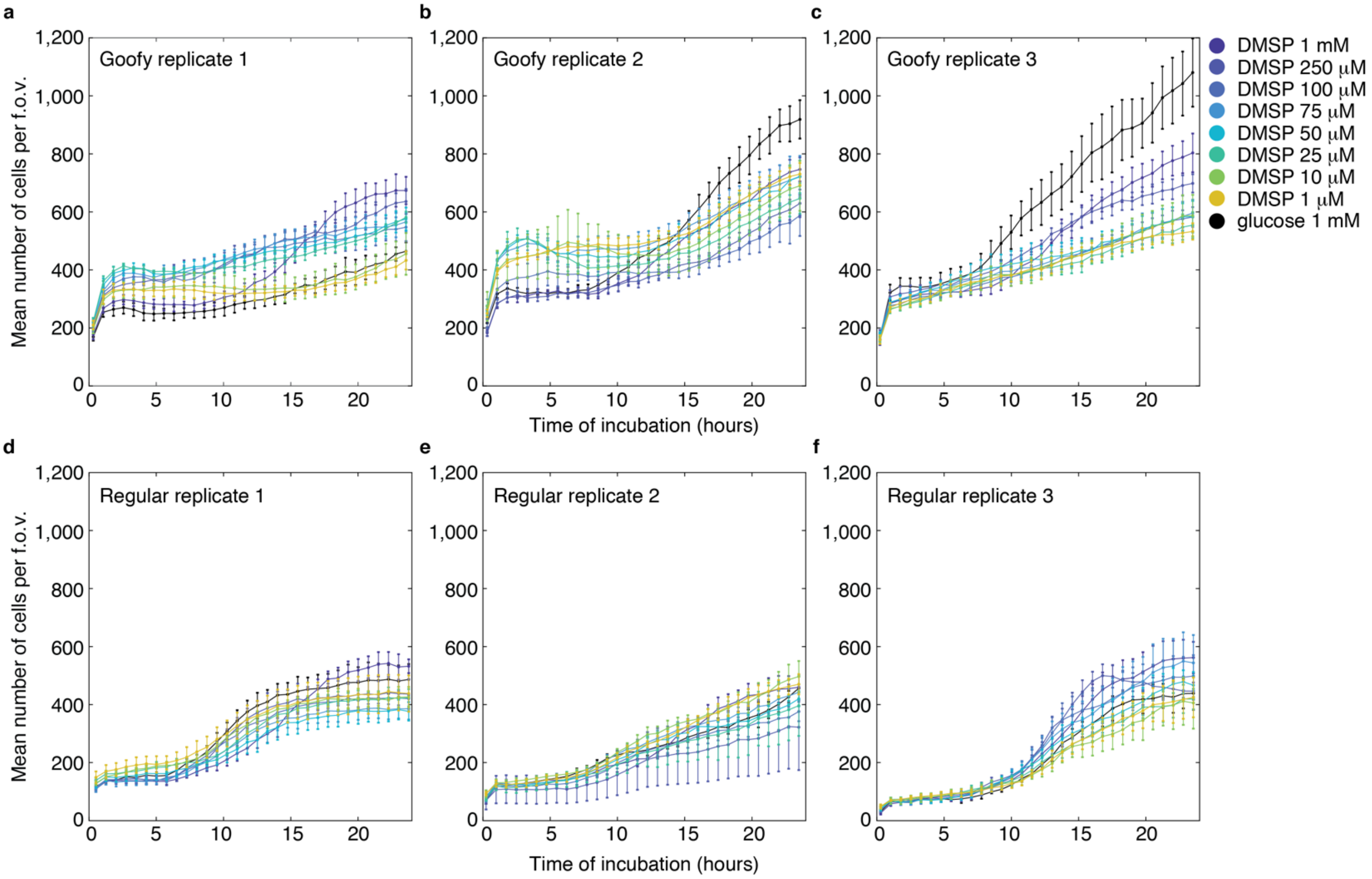

Supplementary Fig. 4 Number of cells in time-lapse DMSP experiments performed in microfluidic chips (all replicate experiments). Mean number of cells per field of view (f.o.v.; 200 μm × 200 μm) in time-lapse DMSP experiments. Images were acquired at seven different fields of view per observation chamber (Fig. 1c) except strain Regular replicate experiment 3 (**f**), in which six fields of view were taken. Only cells that passed the YFP intensity threshold were included in the analysis. The large increase in cell number between the first and second time points is likely attributed to cells settling into the field of view due to gravity. On average, the number of cells in a field of view at the second time point was 218 ± 120 (mean ± s.d.) across replicate experiments. Replicate experiments correspond to those presented in Supplementary Fig. 6. Data points and error bars represent mean ± s.d. across seven (or six for **f**) fields of view.

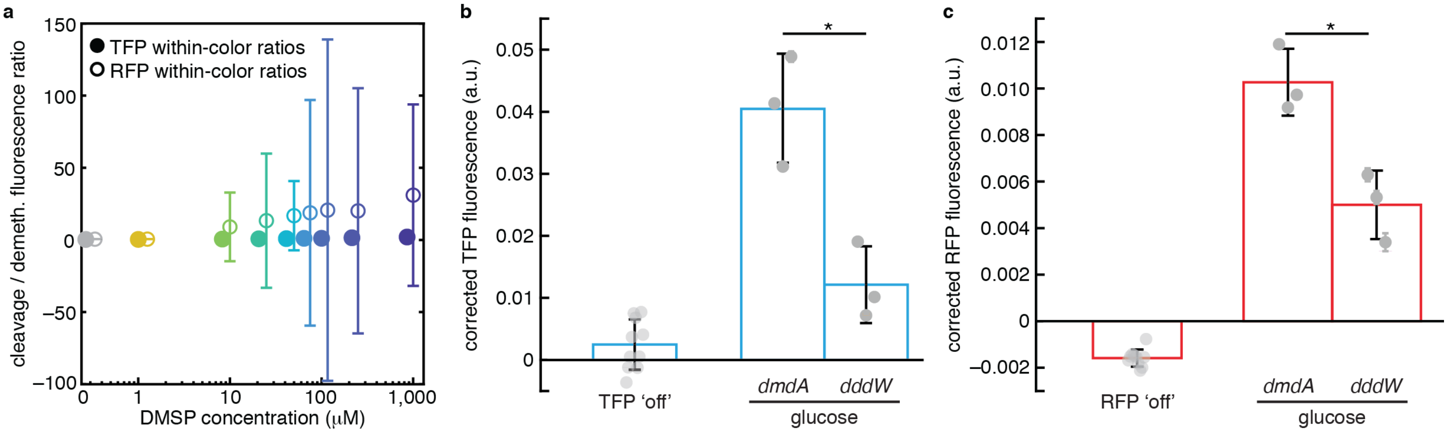

Supplementary Fig. 5 Within-color comparisons of demethylation (*dmdA*) and cleavage (*dddW*) pathway reporters. **a** Cleavage-to-demethylation fluorescence ratios were calculated within each color (TFP/TFP (●) or RFP/RFP (○)) similarly to Fig. 3. At ≥10 μM DMSP, the variability amongst replicate experiments resulted in large variance (error bars) for RFP within-color ratios (though not TFP, probably due to the relatively small signal values of TFP (Supplementary Fig. 6)). This result prevented within-color comparisons of pathway reporters except in glucose (**b**,**c**) and 1 μM DMSP (similar values). Thus, across-color ratio calculation, which enabled comparisons of pathway expression within the same experiment, was employed in our study for consistency (Fig. 3). Error bars represent the variance of the cleavage-to-demethylation ratio calculated using Eq. S6 (Supplementary Note 3; $n = 3$ for each reporter). **b** and **c** Average baseline fluorescence of demethylation and cleavage reporters incubated in glucose (cells aggregated over three time points, ~2.5–4h). In glucose, baseline demethylation expression was significantly higher than that of cleavage (two-tailed *t*-tests; * $p \leq 0.05$; ratios 0.3 in TFP, **b**; 0.5 in RFP, **c**). Importantly, this higher baseline demethylation expression was conserved in both within-color and across-color ratios (0.15–1.0; Fig. 3). Fluorescence signals in glucose were significantly higher than the theoretical 'off' intensities of TFP ($2.5 \pm 4.1 \times 10^{-3}$ a.u.) and RFP ($-1.6 \pm 0.37 \times 10^{-3}$ a.u.), indicating that *dmdA* and *dddW* are expressed at a baseline level even in the absence of DMSP (two-tailed *t*-tests, $p \leq 0.01$). Some theoretical 'off' intensities were slightly overcorrected to below zero due to background subtraction and spectral leakage correction (Supplementary Note 1). Each grey symbol represents a replicate experiment (symbols and error bars = mean ± s.e.m. of fluorescence signals of cells), and each bar represents the average of replicate experiments (bars and error bars = mean ± s.d. of replicate experiments, $n = 3$).

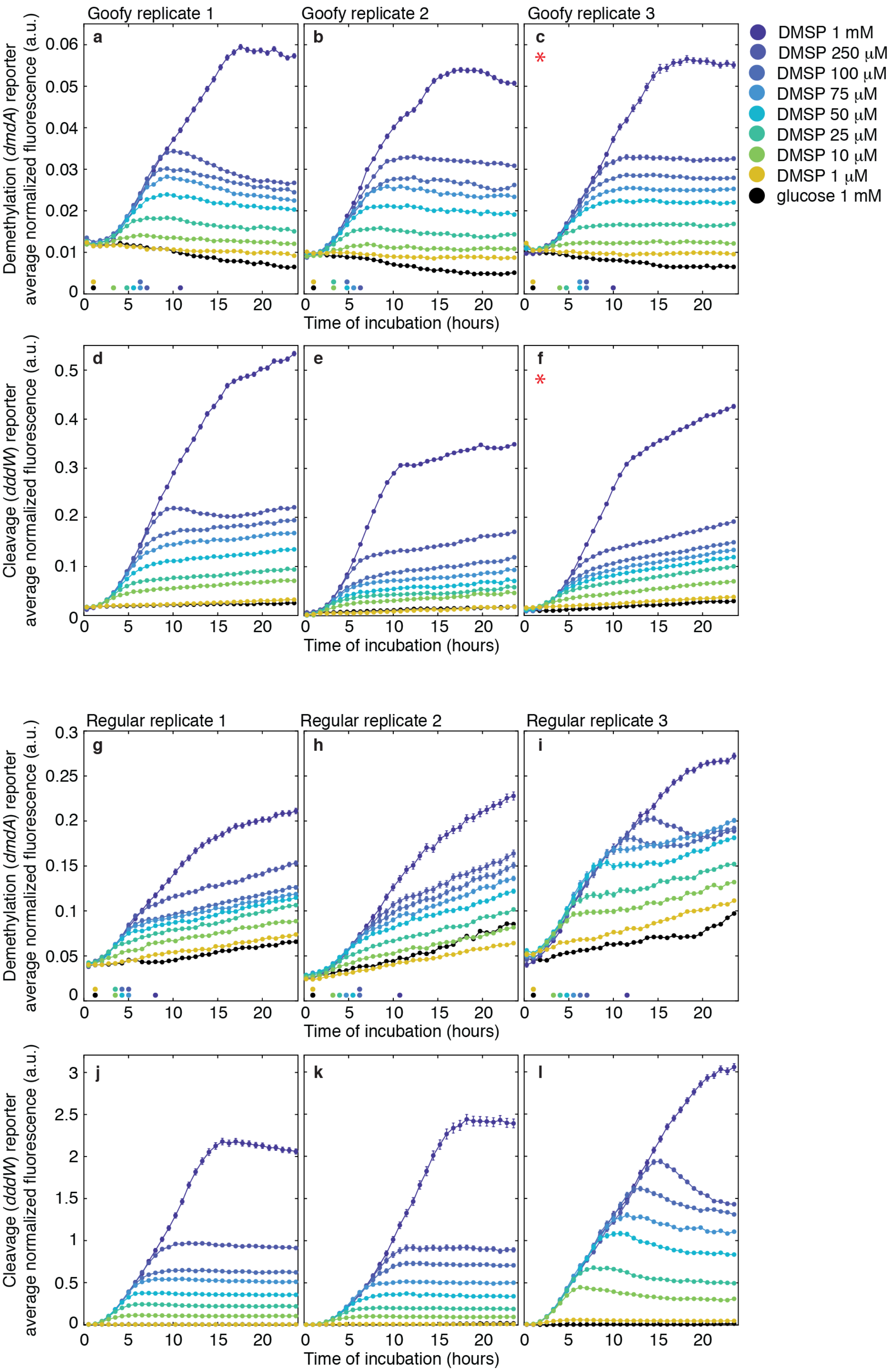
Goofy replicate 1
Goofy replicate 2
Goofy replicate 3
a
b
c
*
DMSP 1 mM
DMSP 250 μM
DMSP 100 μM
DMSP 75 μM
DMSP 50 μM
DMSP 25 μM
DMSP 10 μM
DMSP 1 μM
glucose 1 mM
Demethylation (dmdA) reporter
average normalized fluorescence (a.u.)
Time of incubation (hours)
d
e
f
*
Cleavage (dddW) reporter
average normalized fluorescence (a.u.)
Time of incubation (hours)
Regular replicate 1
Regular replicate 2
Regular replicate 3
g
h
i
Demethylation (dmdA) reporter
average normalized fluorescence (a.u.)
Time of incubation (hours)
j
k
l
Cleavage (dddW) reporter
average normalized fluorescence (a.u.)
Time of incubation (hours)

Supplementary Fig. 6 Demethylation and cleavage pathway expression measurements in time-lapse DMSP experiments performed in microfluidic chips (all replicate experiments). Results from all replicate experiments (*n* = 3 for strain Goofy (**a–f**); *n* = 3 for strain Regular (**g–l**)). In strain Goofy, *dmdA* was reported by RFP (**a–c**) and *dddW* by TFP (**d–f**). In strain Regular, *dmdA* was reported by TFP (**g–i**) and *dddW* by RFP (**j–l**). The time points of mid-exponential in *dmdA* expression, used for cleavage-to-demethylation ratio calculation (shown in Fig. 3), are represented as colored dots at the appropriate time points on the x-axis in demethylation pathway plots (**a–c**, **g–i**). Red asterisks (*) identify the replicate experiment shown in Fig. 2 (**c**,**f**). While the magnitudes of end-point fluorescence intensities, and the timepoints of mid-exponential and saturation of fluorescence kinetics curves, were not the same across replicate experiments and strains, other aspects (*e.g.*, concentration-dependence of end-point fluorescence intensities, and the preservation of slopes of the fluorescence curves across concentrations) were consistent (further discussion in Supplementary Note 2). Data points and error bars represent mean ± s.e.m. of fluorescence signals of cells.

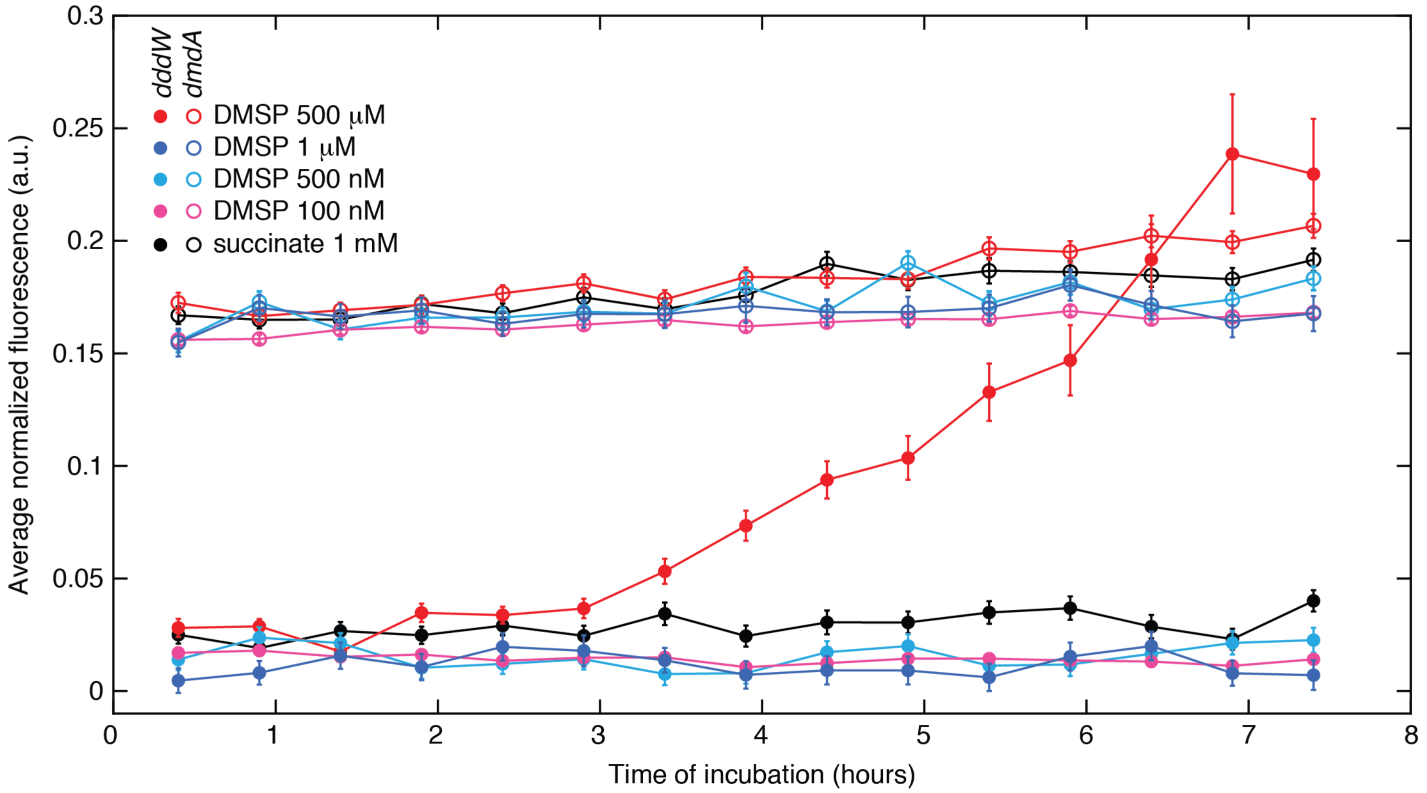

Supplementary Fig. 7 Fluorescence response to DMSP concentrations below 1 μM was undetectable in the microfluidic chip experimental setup. Lower DMSP concentration experiment (≤1 μM): *R. pomeroyi* reporter strain Regular was incubated with succinate (negative control) or DMSP (at 100 nM, 500 nM, 1 μM, or 500 μM) in observation chambers of a microfluidic chip in the presence of 50 μg/ml kanamycin. TFP (*dmdA* reporter; open circles) and RFP (*dddW* reporter; closed circles) fluorescence of each cell were normalized by its own YFP intensity (constitutive expression). Each experimental condition was imaged at 4–10 different positions, with each field of view containing 25–200 cells that passed the constitutive YFP fluorescence intensity threshold. No cell division was observed over the experimental duration. Fluorescence signal upregulation above baseline was either absent, or not detectable, in DMSP concentrations at and below 1 μM compared to succinate (two-tailed *t*-test at 6.4 h, $p > 0.01$). Data points and error bars represent mean ± s.e.m. of normalized fluorescence intensities of cells. This represents the only experiment in which succinate was used as negative control. Succinate, which did not elicit a fluorescence reporter response (Supplementary Fig. 3) and possesses a molecular weight that is similar to DMSP, was initially used as negative control in some early experiments. All other experiments presented in this manuscript used glucose as the negative control.

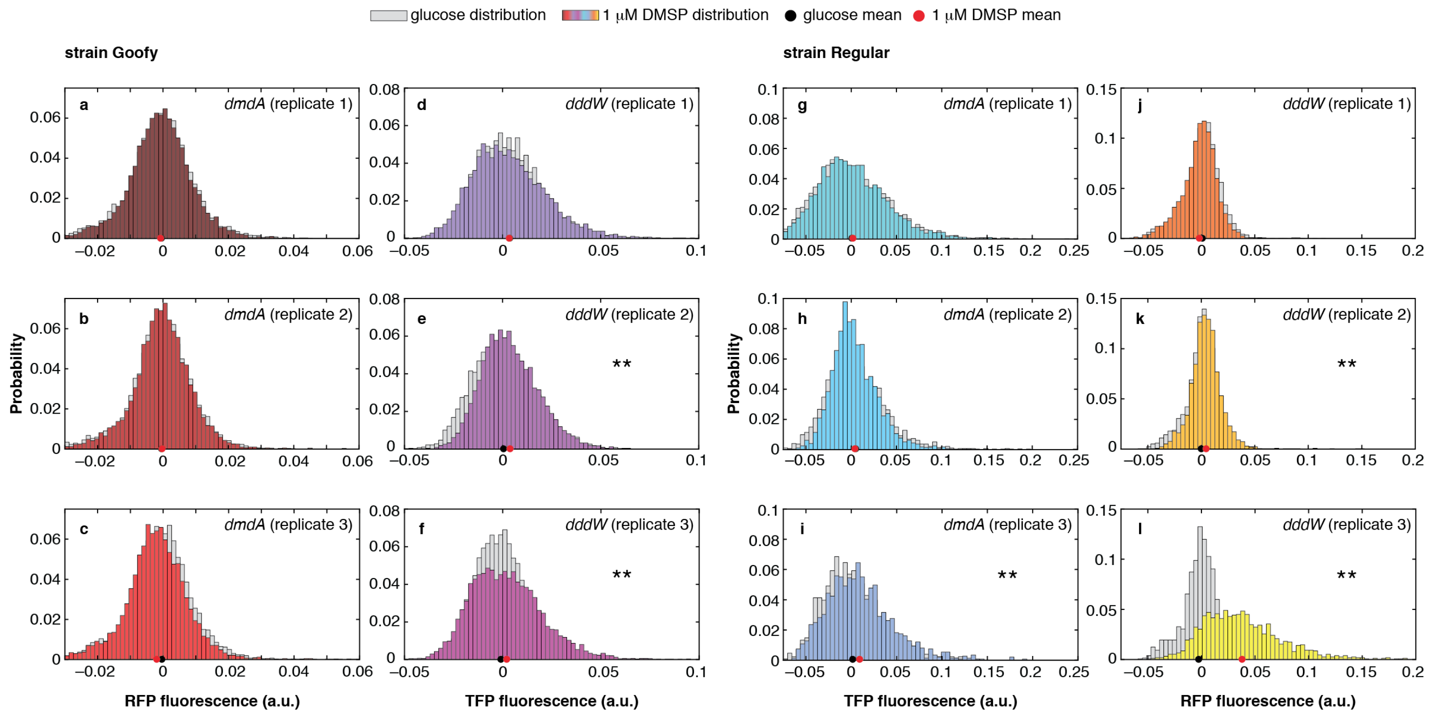

Supplementary Fig. 8 Onset of *dmdA* and *dddW* upregulation at 1 μM DMSP in microfluidic experiments. Probability distributions of fluorescence signals of *dmdA* (**a–c, g–i**) or *dddW* (**d–f, j–l**) reporters (strain Goofy (**a–f**) or Regular (**g–l**)) incubated with 1 mM glucose or 1 μM DMSP in microfluidic chip experiments (cells aggregated across three time points, ~2.5–4 h). Mean fluorescence of the first time point was subtracted from the fluorescence of each cell in appropriate color channels. Two-tailed *t*-test was performed on fluorescence data within each replicate experiment to determine statistically significant upregulation of *dmdA* or *dddW* in DMSP compared to glucose (glucose, n = 1,284–7,191 cells; DMSP, n = 1,488–9,362 cells; **, significant upregulation with $p < 0.01$). Only one (**i**) out of six replicate experiments showed significant upregulation of the demethylation pathway (*dmdA*) at 1 μM DMSP compared to glucose, while at higher concentrations (≥10 μM) all experiments exhibited upregulation of *dmdA* (Supplementary Fig. 6). These results suggest that 1 μM approximates the threshold DMSP concentration above which bacteria increase *dmdA* gene expression beyond baseline levels.

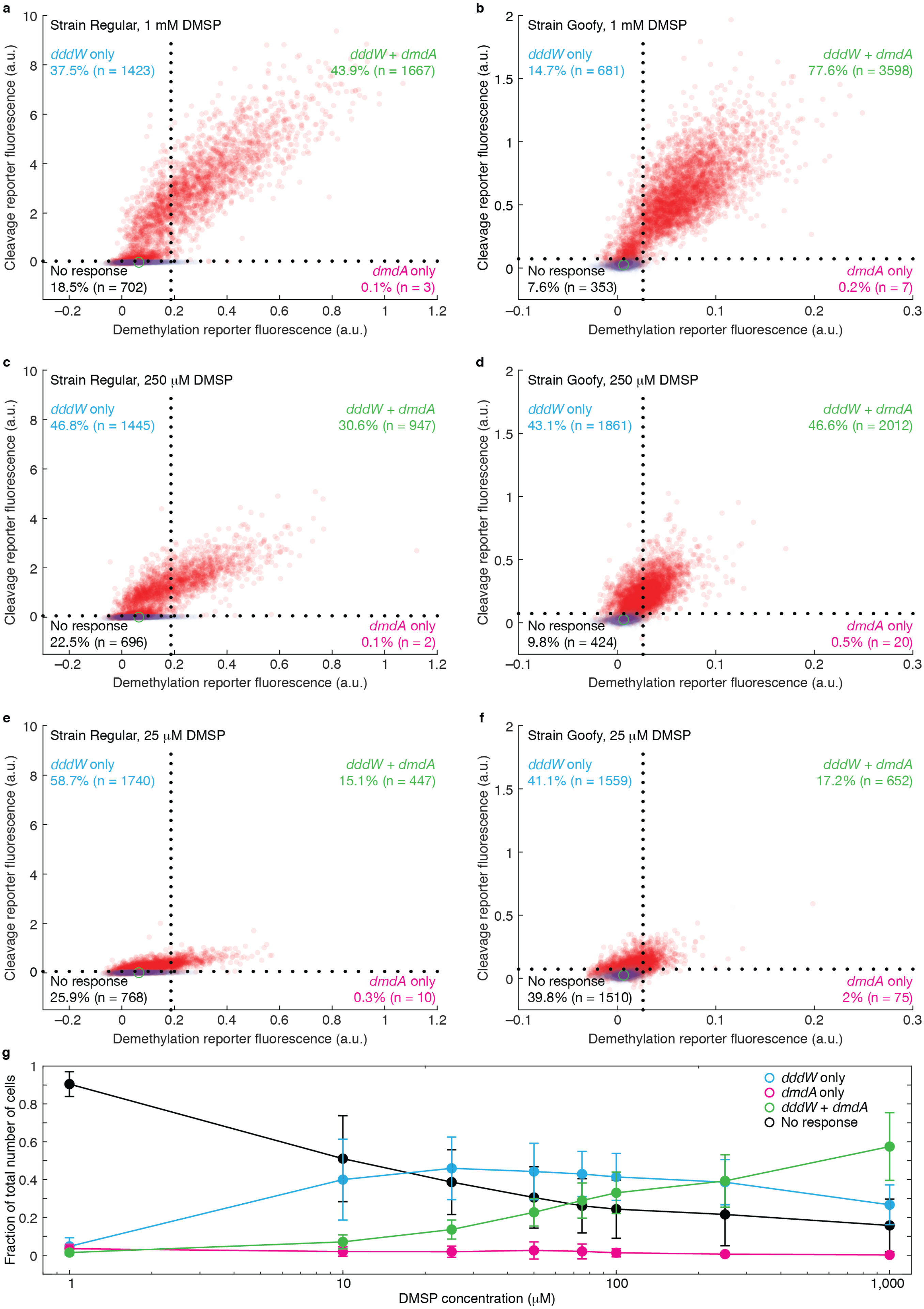
a
Strain Regular, 1 mM DMSP
dddW only
37.5% (n = 1423)
dddW + dmdA
43.9% (n = 1667)
No response
18.5% (n = 702)
dmdA only
0.1% (n = 3)
b
Strain Goofy, 1 mM DMSP
dddW only
14.7% (n = 681)
dddW + dmdA
77.6% (n = 3598)
No response
7.6% (n = 353)
dmdA only
0.2% (n = 7)
c
Strain Regular, 250 μM DMSP
dddW only
46.8% (n = 1445)
dddW + dmdA
30.6% (n = 947)
No response
22.5% (n = 696)
dmdA only
0.1% (n = 2)
d
Strain Goofy, 250 μM DMSP
dddW only
43.1% (n = 1861)
dddW + dmdA
46.6% (n = 2012)
No response
9.8% (n = 424)
dmdA only
0.5% (n = 20)
e
Strain Regular, 25 μM DMSP
dddW only
58.7% (n = 1740)
dddW + dmdA
15.1% (n = 447)
No response
25.9% (n = 768)
dmdA only
0.3% (n = 10)
f
Strain Goofy, 25 μM DMSP
dddW only
41.1% (n = 1559)
dddW + dmdA
17.2% (n = 652)
No response
39.8% (n = 1510)
dmdA only
2% (n = 75)
Cleavage reporter fluorescence (a.u.)
Demethylation reporter fluorescence (a.u.)
g
Fraction of total number of cells
DMSP concentration (μM)
dddW only
dmdA only
dddW + dmdA
No response

Supplementary Fig. 9 Upregulation of demethylation (*dmdA*) and cleavage (*dddW*) at the single-cell level. **a–f**, Fluorescence of *dmdA* and *dddW* reporters at ~23 h (time point 30) from representative microfluidic chip experiments are shown (also in Supplementary Fig. 6): replicate experiment 1 of strain Regular (**a**,**c**,**e**) and replicate experiment 1 of strain Goofy (**b**,**d**,**f**). Each data point represents fluorescence signals of a single cell. Blue dots represent cells that have been incubated with 1 mM glucose, while red dots represent cells that have been incubated with DMSP at 1 mM (**a**,**b**), 250 μM (**c**,**d**), or 25 μM (**e,f**). Dotted lines mark two standard deviations from the mean (green circle) of the glucose population, representing cutoffs for defining the four populations: cells that upregulated (1) *dddW* only (cyan), (2) *dmdA* only (magenta), (3) both pathways (green), and (4) neither pathways (no response, black). **g**, Fractions of total number of cells at ~23 h (time point 30) in each of the four populations, averaged across all replicate experiments of both reporter strains (Goofy and Regular). At DMSP concentrations ranging between 10–100 μM, the '*dddW* only' population (cyan) represented the majority of responsive cells. At DMSP concentrations ranging from 250 μM–1 mM, cells that upregulate both pathways (green) formed the majority. Data points and error bars represent mean ± s.d. of replicate experiments (n = 6). Heterogeneity in DMSP degradation gene upregulation is discussed in Supplementary Note 2.

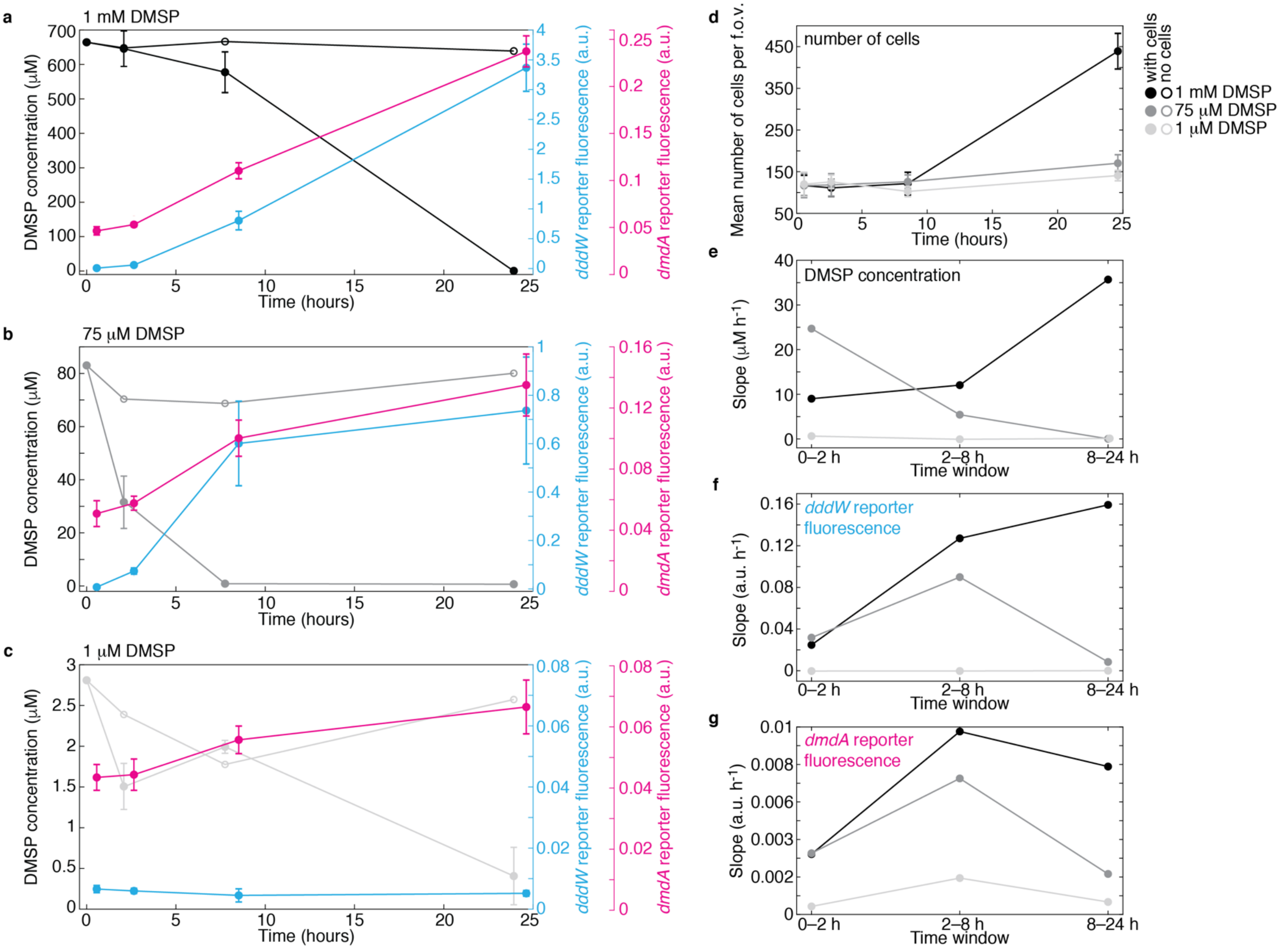

Supplementary Fig. 10 Concentrations of DMSP in time-lapse experiments decrease over time due to cell uptake. **a–c** DMSP concentration measurements in a large-volume (8 ml) experiment with engineered *R. pomeroyi* (strain Regular) incubated with 1 mM DMSP (**a**), 75 μM DMSP (**b**), or 1 μM DMSP (**c**). Small differences between measured and expected initial concentrations are attributed to experimental or measurement errors. Samples were taken for DMSP concentration measurements (left y-axis, black) and fluorescence microscopy (right y-axes, cyan and magenta) at approximately 0, 2, 8, and 24 h after the start of incubation. Consistent with our microfluidic chip experiments, both pathways increased expression when bacteria were incubated with 75 μM and 1 mM DMSP, but 1 μM DMSP led to little or no response in either pathway. Data points and error bars represent mean ± s.d. of three biological replicates for DMSP concentration measurements and fluorescence intensities. Specifically, mean fluorescence intensities of cells within a field of view, then across fields of views, were calculated before averaging across the three biological replicates (n = 90–151 average number of cells per field of view at 0 h; seven fields of views per condition at 0 h; two fields of views per condition at subsequent time points). Each cell's fluorescence signals were normalized by its own constitutive-YFP signal. **d** Mean number of cells per field of view (f.o.v.; 200 μm × 200 μm), counted in images taken for fluorescence intensity quantification at each sampling time point. Growth was observed in all DMSP concentrations. At 1 mM DMSP, a burst of growth during the last two time

points (between 8 and 24 h after start of incubation), coincided with the largest DMSP concentration decrease (**a**). Data points and error bars represent mean ± s.d. across three biological replicates of average number of cells per f.o.v. **e–g** Rates of change in DMSP concentration (**e**), cleavage (*dddW*) reporter fluorescence (**f**), and demethylation (*dmdA*) reporter fluorescence (**g**). Slopes between pairwise time points were calculated for each DMSP concentration condition. The 1 mM condition (black) is marked by an initial period (0–8 h) of slow DMSP concentration decay (**e**) but rapid pathway upregulation (**f**,**g**). This initial period is followed by fast DMSP uptake (8–24 h; **e**) coinciding with rapid increase in cell number (**d**) and continued increase in pathway expression (**f**,**g**). In contrast, the 75 μM condition (dark grey) is marked by a rapid initial decline in DMSP concentration during the first time points (0–2 h; **e**), followed by a period of fast pathway expression increase (2–8 h; **f**,**g**), which plateau at the same time as DMSP is depleted (8–24 h; **b**). Differences in rates of DMSP uptake, growth, and pathway expression between 75 μM and 1 mM DMSP conditions point to potentially different physiological adaptations corresponding to low and high nutrient conditions.

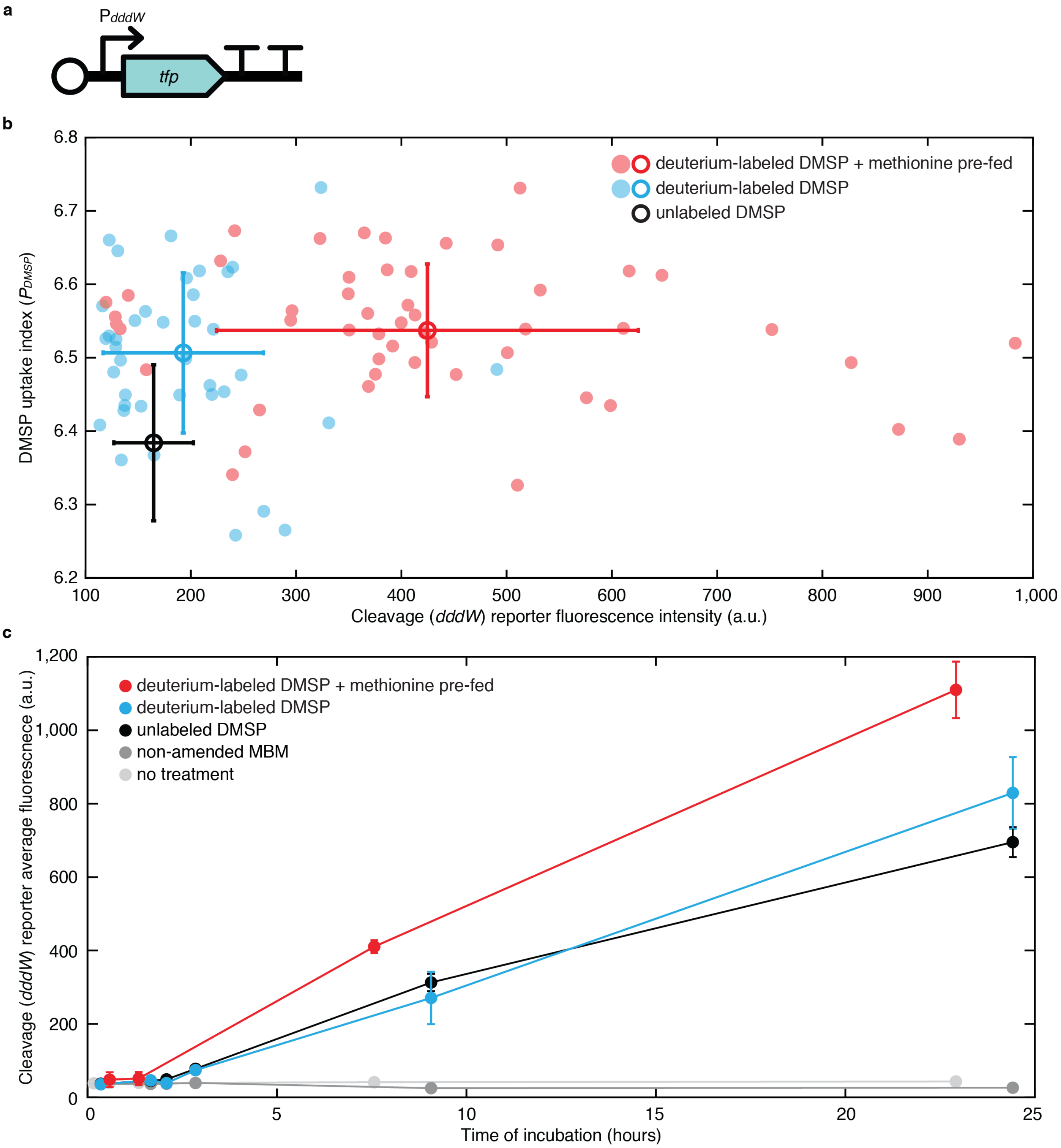
a
P$_{dddW}$
tfp
b
deuterium-labeled DMSP + methionine pre-fed
deuterium-labeled DMSP
unlabeled DMSP
DMSP uptake index (P_{DMSP})
Cleavage (dddW) reporter fluorescence intensity (a.u.)
6.8
6.7
6.6
6.5
6.4
6.3
6.2
100
200
300
400
500
600
700
800
900
1,000
c
deuterium-labeled DMSP + methionine pre-fed
deuterium-labeled DMSP
unlabeled DMSP
non-amended MBM
no treatment
Cleavage (dddW) reporter average fluorescnece (a.u.)
Time of incubation (hours)
1,200
1,000
800
600
400
200
0
5
10
15
20
25

Supplementary Fig. 11 Cells satiated in sulfur maintain DMSP uptake but increase cleavage pathway expression. **a** The P_{dddW}::mTFP1 single-color *R. pomeroyi* reporter strain (cleavage pathway promoter-fusion with TFP) was used to avoid spectral interference with Raman microspectroscopy measurements. **b** Raman microspectroscopy measurements were performed at 5.5 h after incubation initiation. The reporter strain (**a**) was incubated with 1 mM deuterium-labeled DMSP ([$^2H_6$]-DMSP), either with or without prior exposure to 10 mM L-methionine (an alternative sulfur source to DMSP). All DMSP and methionine were dissolved in marine basal medium (MBM). The DMSP uptake index, P_{DMSP}, was calculated as the ratio of integrated intensities at Raman spectrum regions between the Raman wavenumbers 2040 and 2300 cm^{-1} (C-D peak) and between 2400 and 2450 cm^{-1} (reference region where background intensity was low) (Methods). Each filled-circle data point represents the P_{DMSP} value and the background-subtracted, raw TFP fluorescence signal of a single cell, measured by the Raman microspectroscopy setup (Methods) (n = 43 cells treated with deuterium-labeled DMSP only (cyan); n = 50 cells pre-fed with methionine and treated with deuterium-labeled DMSP (red)). For clarity of presentation, individual cell data points for unlabeled DMSP condition are not shown. Empty circles and error bars represent mean ± s.d. of fluorescence (horizontal) and DMSP uptake index (vertical) of cells in each experimental condition. **c** Fluorescence response of cells incubated with deuterium-labeled DMSP (red and cyan), unlabeled DMSP (black), non-carbon amended MBM (dark grey), or no treatment (light grey) in observation chambers, monitored over time by microscopy (start of incubation at 0h). All conditions except 'no treatment' contained a final concentration of 1% methanol (solvent in which [$^2H_6$]-DMSP was dissolved). At each time point, seven images were taken at different positions within each observation chamber (n = 330 ± 325 (mean ± s.d.) cells per image). Following image segmentation and background subtraction (Methods), the mean cellular fluorescence was calculated for each image, then averaged across images. Results confirmed that DMSP, unlabeled (black) or deuterium-labeled (cyan), elicit the same fluorescence response. Pre-feeding with methionine (red) led to faster upregulation and higher fluorescence signals of the cleavage (*dddW*) reporter cells compared to control cells (*i.e.*, not pre-fed with methionine). Data points and error bars represent mean ± s.d. of average cellular fluorescence of seven images.

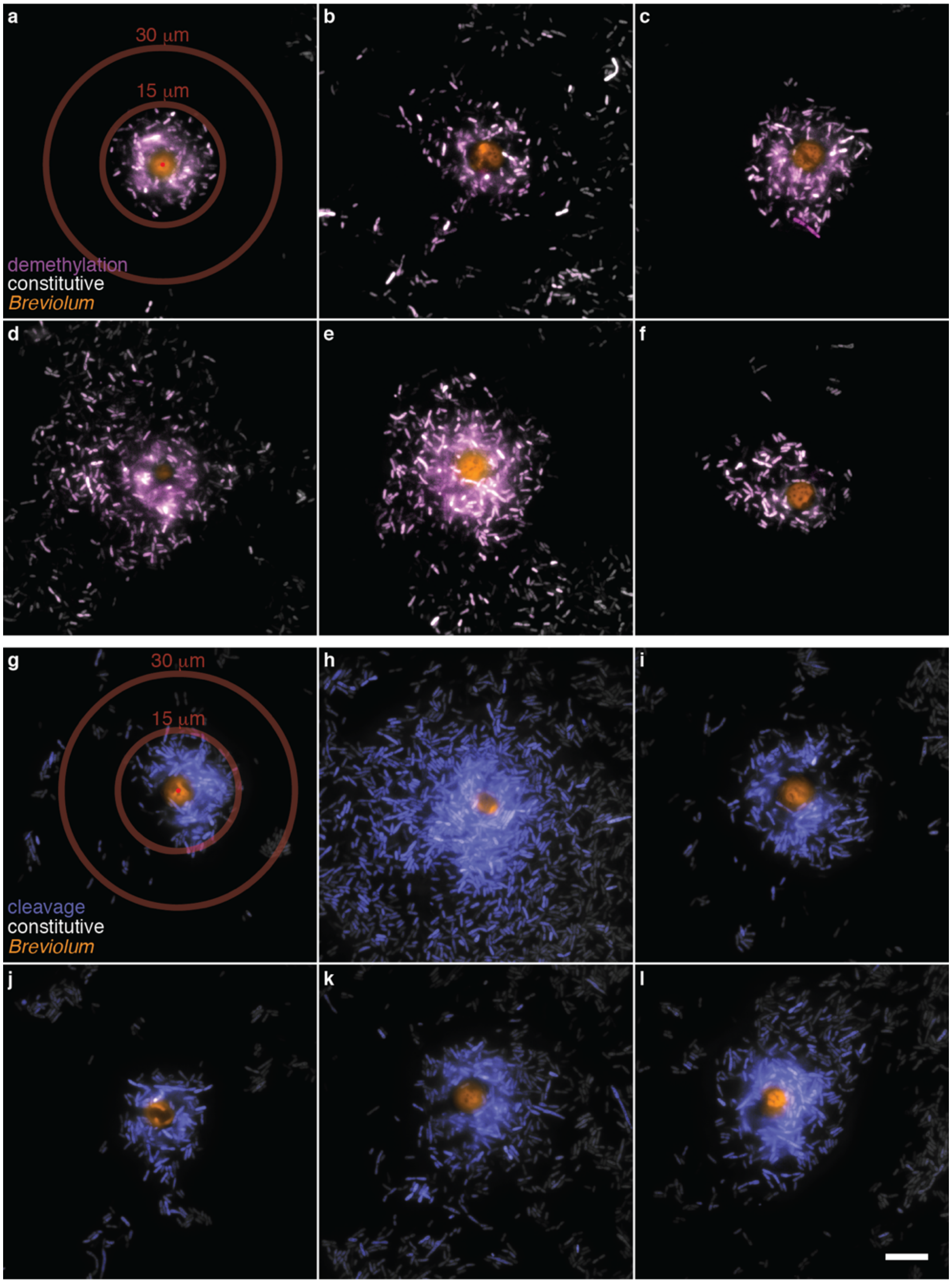

Supplementary Fig. 12 Images of phytoplankton-bacteria co-incubation experiment. Representative images of the agarose pad co-incubation experiment (also shown in Fig. 4) with DMSP-producing phytoplankton, *Breviolum* CCMP2459, surrounded by *R. pomeroyi* bacteria fluorescently reporting *dmdA* (**a–f**, strain Regular; out of 15 total images) or *dddW* (**g–l**, strain Goofy; out of 18 total images) gene expression. Fluorescence signals are false-colored orange for photosynthetic pigments of phytoplankton, white for YFP (constitutive expression), magenta for *dmdA* reporter (TFP; **a–f**), and blue for *dddW* reporter (TFP; **g–l**). Representative concentric rings (widths of 20 pixels = 1.6 μm), used to bin distances from the center of *Breviolum* cells (red dots; **a**,**g**) for quantification of fluorescence signals, are shown for distances 15 and 30 μm. Scale bar, 15 μm.

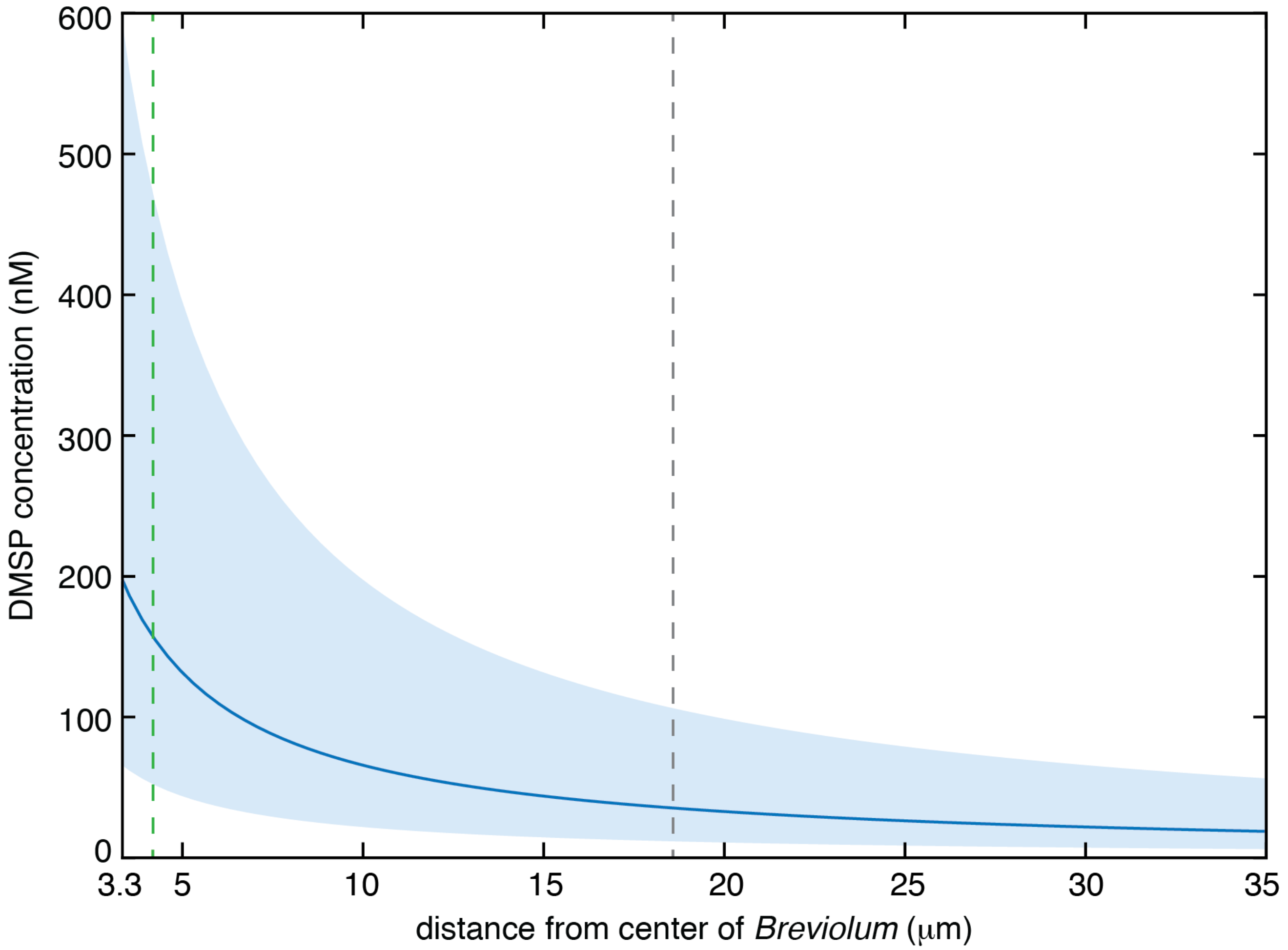

Supplementary Fig. 13 Model of DMSP concentration in a phycosphere. Concentration profile of DMSP within a phycosphere predicted using Eq. S7 (Supplementary Note 4). The dark blue line shows modeled DMSP concentrations at distances (r) from the center of a *Breviolum* cell, with a radius of 3.3 μm (average radius in our co-incubation experiment; lower x-axis limit). Modeled DMSP concentration at the surface of the cell (r = 3.3 μm) was 197 nM. Green dotted line marks the distance of the first concentric ring at which bacterial fluorescence was quantified (r = 4.2 μm; modeled DMSP concentration = 157 nM). Grey dotted line represents the distance within the phycosphere beyond which bacterial fluorescence signals were observed to return to baseline levels (r = 18.6 μm; modeled DMSP concentration = 35 nM). Shaded regions show two further cases in which modeled DMSP concentrations are 3-fold greater (upper boundary of the shaded region) or 3-fold smaller (lower boundary) than in the baseline case.

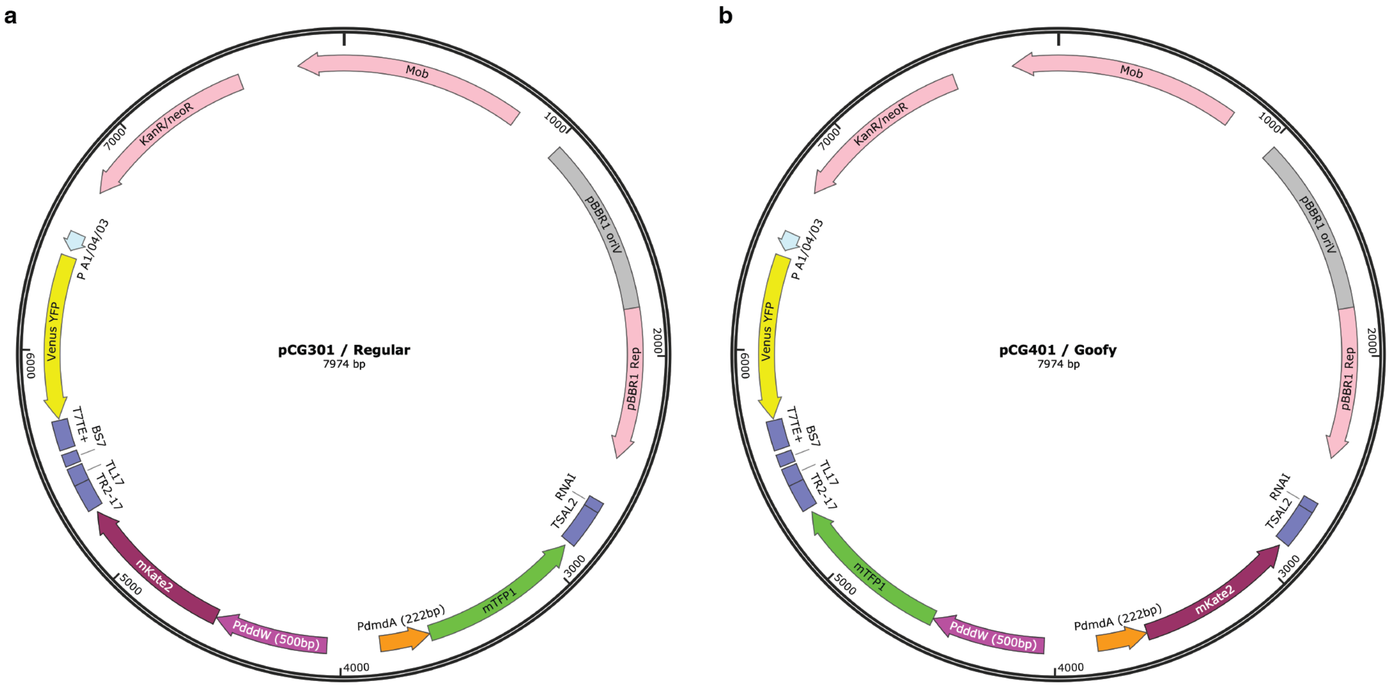

Supplementary Fig. 14 Plasmid maps of tricolor reporter constructs, pCG301 (**a**, strain Regular) and pCG401 (**b**, strain Goofy).

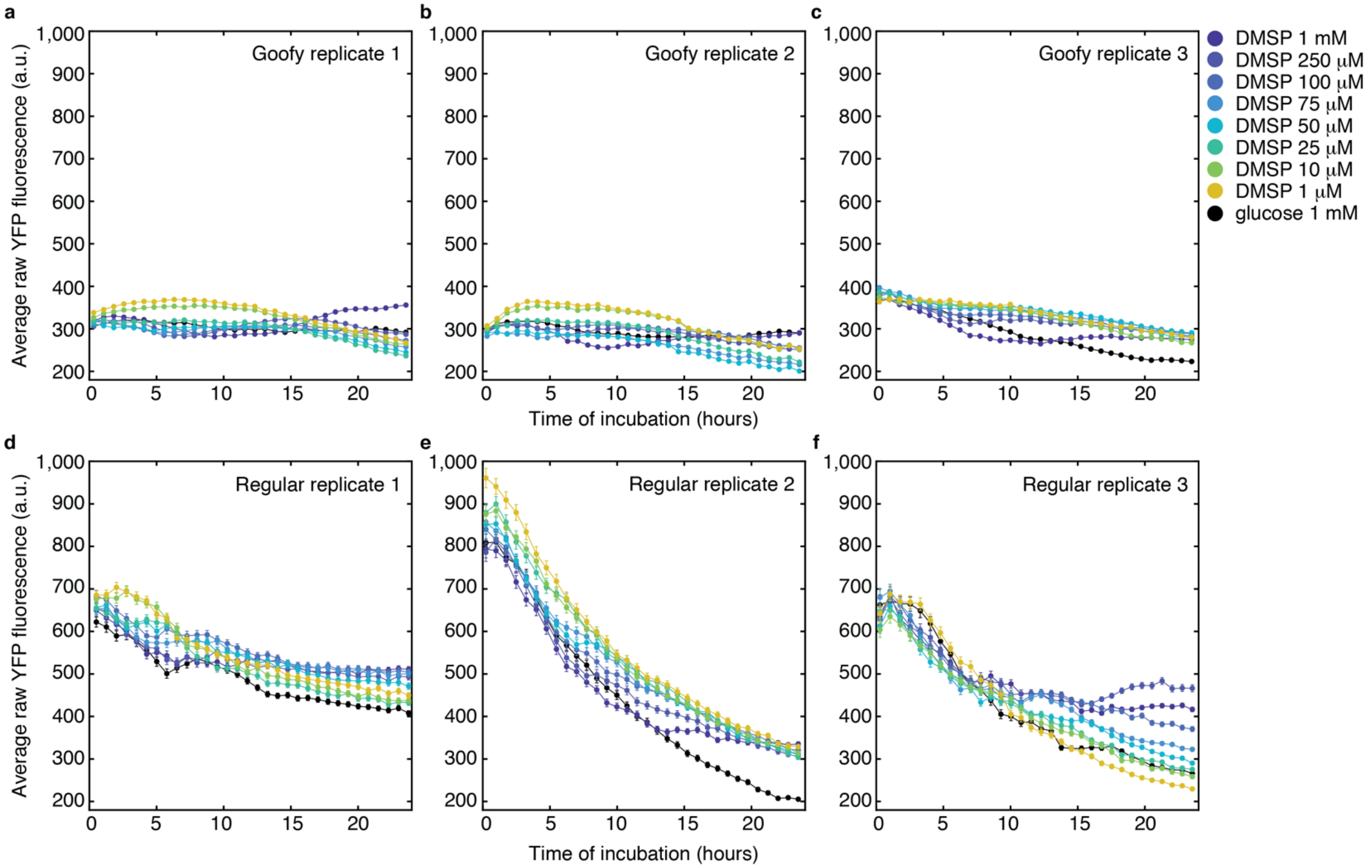

Supplementary Fig. 15 YFP fluorescence in time-lapse DMSP experiments performed in microfluidic chips (all replicate experiments). Replicate experiments correspond to those presented in Supplementary Fig. 6. Background subtraction, spectral leakage correction, and YFP intensity thresholding (50 a.u.) were performed. YFP signals decreased over the duration of experiments, which may be due to a number of factors including plasmid-loss by bacteria and fluorescence signal dilution due to cell division. Data points and error bars represent mean ± s.e.m. of fluorescence signals of cells.

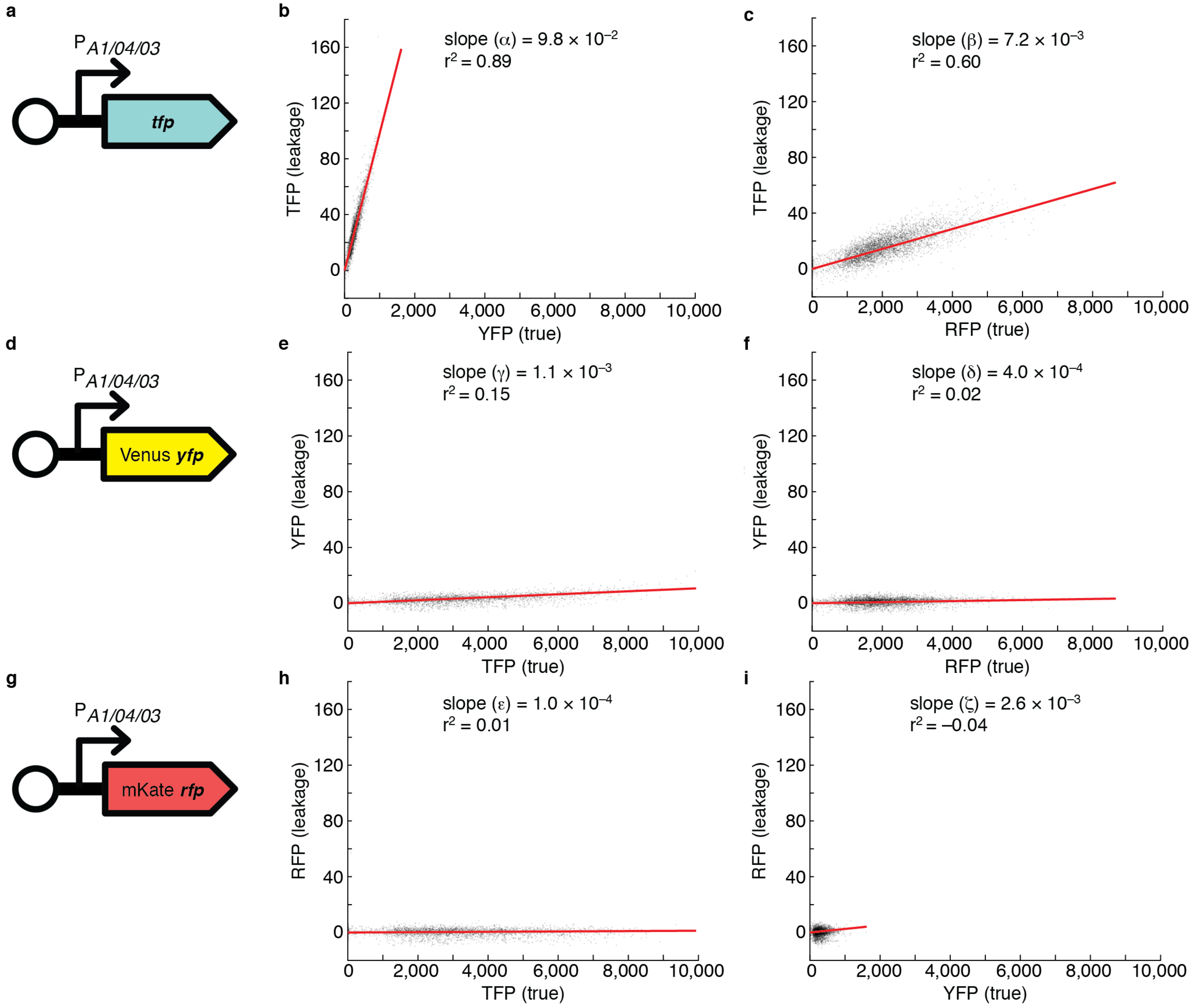

Supplementary Fig. 16 Calculation of spectral leakage correction matrix, *B*, with constitutively fluorescent single-color control strains. Each constitutively fluorescent single-color control strain of *R. pomeroyi* (**a**, $P_{A1/04/03}$::mTFP1; **d**, $P_{A1/04/03}$::YFP; **g**, $P_{A1/04/03}$::mKate2) was imaged in its appropriate color channel ('true') and the two inappropriate color channels ('leakage') (*n* = 10 biological replicates per strain; data point = one cell in **b–c**, **e–f**, **h–i**). Non-fluorescent cells were eliminated from analyses by applying minimum intensity thresholds in the true fluorescence channels (Supplementary Note 1). Only 10% of cells, randomly sampled across ten biological replicates, are plotted for clarity. In images of tricolor reporter strains (Regular or Goofy), recorded fluorescence signals are sums of 'true' and 'leakage' signals (Eq. S1–3). Specifically, recorded teal fluorescence ($\hat{t}$) includes leakage from YFP (αy; **b**) and RFP (βr; **c**); recorded yellow fluorescence ($\hat{y}$) includes leakage from TFP (γt; **e**) and RFP (δr; **f**); recorded red fluorescence ($\hat{r}$) includes leakage from TFP (εt; **h**) and YFP (ζy; **i**). Slopes of linear regressions (α,β,γ,δ,ε,ζ) were used to generate the spectral leakage correction matrix, *B*, which was used for quantification of true fluorescence signals of tricolor reporter strains (Supplementary Note 1).

Supplementary Tables

Supplementary Table 1

Fluorescent strains constructed in *R. pomeroyi* DSS-3 for this study.

**Strain / Plasmid**	**Description**	**GenBank Accession #**
Regular / pCG301	tricolor reporter (P_{dmdA}::mTFP1, P_{dddW}::mKate2, $P_{A1/04/03}$::YFP)	MN744962
pCG302	single-color reporter (P_{dmdA}::mTFP1)	MN744963
pCG303	single-color reporter (P_{dddW}::mKate2)	MN744964
Goofy / pCG401	tricolor reporter (P_{dmdA}::mKate2, P_{dddW}::mTFP1, $P_{A1/04/03}$::YFP)	MN744965
pCG402	single-color reporter (P_{dmdA}::mKate2)	MN744966
pCG403	single-color reporter (P_{dddW}::mTFP1)	MN744967
pCG101	single-color constitutive control ($P_{A1/04/03}$::mKate2)	MN744959
pCG102	single-color constitutive control ($P_{A1/04/03}$::YFP)	MN744960
pCG103	single-color constitutive control ($P_{A1/04/03}$::mTFP1)	MN744961
pZS2-200	pZS2-123 with replaced promoters (P_{dmdA}, P_{dddW}, P_{lac}) for cloning aid only	MN744968

Supplementary Table 2

Sequences of all oligonucleotide primers used in this study (in accompanying Excel sheet).

Supplementary Table 3

Plasmids externally obtained and used in this study.

Plasmid	Description	Source / Reference
pZS2-123	DNA architecture of tricolor reporters; source of Venus YFP and terminators	AddGene #26598 (Reference: 1)
pBBR1MCS-2	vector backbone (Kan^R)	Provided by Prof. M.E. Kovach (Reference: 2)
pXGFPC-2 P_{lac}::mKate2	source of mKate2 and $P_{A1/04/03}$ modified *lac* promoter	Provided by G. D'Souza (Reference: 3)
pXGFPC-2 P_{lac}::mTFP1	source of mTFP1	Provided by G. D'Souza (Reference: 3)
pRK415	source of *lac* promoter that is weakly active in DSS-3	Provided by Prof. C.R. Reisch (Reference: 4, 5)
pKR600	helper strain (*E. coli*) for triparental mating (Cam^R)	Provided by Prof. C.R. Reisch (Reference: 6)

Supplementary Notes

Supplementary Note 1: Image analysis for cellular fluorescence quantification

1.1. Cell segmentation, thresholding, and generation of ‘cell mask’ and ‘background mask’

Analysis of fluorescence images was performed in MATLAB (MathWorks) using an automated image segmentation and fluorescence quantification software developed in-house. First, cell-containing pixels were recognized by applying a threshold (determined manually for each replicate experiment) on pixel intensity in phase contrast images, in which cells appear as dark (*i.e.*, low intensity) pixels. Subsequently, cells were segmented by grouping cell-containing pixels that were in contact with each other.

Not all recognized cells were suitable for further analyses due to their orientation or size. Cells that were positioned perpendicular to the glass slide, which contained high intensity pixels in phase contrast images, were not suitable for quantification due to the larger integration distance in the z-direction for fluorescence signals. These perpendicular cells were eliminated from further analyses by applying a threshold (determined manually for each replicate experiment) on the upper quartile pixel intensity within each cell in phase contrast images, whereby cells containing many high intensity (*i.e.*, white) pixels were eliminated from further analyses. Furthermore, thresholds on maximum and minimum size (*i.e.*, number of pixels within a segmented cell; pixel size = 0.2 μm × 0.2 μm) eliminated aggregates of many cells (large size; maximum area threshold = 200 pixels), or other particles (small size; minimum area threshold = 10 pixels). The average size of a cell was 39.0 ± 4.02 pixels (mean ± s.d. of cells in replicate experiments). As a result of thresholding, ~20% of originally recognized cells were eliminated from further analyses. The position of each cell was recorded, and a ‘cell mask’ was generated from each phase contrast image. Appropriate cell segmentation and thresholding were visually inspected and confirmed for each image. In some images, a small offset (*e.g.*, by 1–5 pixels) between ‘cell masks’ and fluorescence images were manually corrected.

Background fluorescence was defined as the average intensity of pixels that do not contain cells, within a fluorescence channel image. Cell-containing areas of ‘cell masks’ were dilated by a radius of 20–30 pixels, and the negatives of these expanded ‘cell masks’ served as ‘background masks’. Representative samples of ‘background masks’ were visually inspected for appropriate background pixel identification. The background intensity for each fluorescence image was calculated as the average intensity of pixels containing no cells, labeled in ‘background masks’.

Cell and background masks were applied to fluorescence images for cellular and background fluorescence intensity quantification. Fluorescence intensity (TFP, YFP, or RFP) of each cell was defined as the mean intensity of pixels contained within a recognized cell. Background fluorescence was subtracted from each cell to account for variation in background signal levels in different images.

1.2. Spectral leakage correction in image processing and analyses

Our tricolor reporter strains (Fig. 1a,b) expressed three fluorescent proteins (TFP, YFP, and RFP) which were chosen for maximum spectral separation. However, the wide excitation and emission spectra of fluorescent proteins, combined with the range of wavelengths that pass through emission filters of filter cubes, led to some fluorescence emission signals leaking into inappropriate channels (*e.g.*, fluorescence emission from YFP leaking into the teal channel). Thus, spectral leakage correction was applied during image processing and analyses for accurate quantification of true fluorescence intensities. True fluorescence intensities expressed by the tricolor reporter strains are represented in the following equations:

$$\hat{t} = \boldsymbol{t} + \alpha y + \beta r \qquad \text{(Eq. S1)}$$

$$\hat{y} = \gamma t + \boldsymbol{y} + \delta r \qquad \text{(Eq. S2)}$$

$$\hat{r} = \varepsilon t + \zeta y + \boldsymbol{r} \qquad \text{(Eq. S3)}$$

where $\hat{t}$, $\hat{y}$, and $\hat{r}$ represent the recorded intensities in teal, yellow, and red channels, respectively. The recorded intensity ($\hat{t}$, $\hat{y}$, or $\hat{r}$) of each fluorescent protein was expressed as a sum of (i) the *true* intensity (t, y, or r) in the color channel that match the fluorescent protein of interest and (ii) *leakage* intensities from the other two fluorescent proteins, which were represented as fractions (α, β, γ, δ, ε, and ζ) of the true intensities of the two auxiliary fluorescent proteins. Equations S1–S3 can also be represented in aggregate:

$$\widehat{\begin{bmatrix} t \\ y \\ r \end{bmatrix}} = B \times \begin{bmatrix} \boldsymbol{t} \\ \boldsymbol{y} \\ \boldsymbol{r} \end{bmatrix} \qquad \text{(Eq. S4)}$$

where

$$B = \begin{bmatrix} 1 & \alpha & \beta \\ \gamma & 1 & \delta \\ \varepsilon & \zeta & 1 \end{bmatrix} \qquad \text{(Eq. S5)}$$

Thus, *B* is the spectral leakage correction matrix that enabled us to solve for the true intensities (t, y, and r) from recorded intensities ($\hat{t}$, $\hat{y}$, and $\hat{r}$).

1.3. Calculation of spectral leakage correction matrix, *B*

To calculate the spectral leakage correction matrix, *B*, the intensities of signals that leaked into inappropriate channels were quantified for each fluorescent protein (Supplementary Fig. 16). Constitutively fluorescent single-color control strains (TFP-, YFP-, or RFP-expressing strains) were grown as described in Methods. These control strains were placed in separate observation chambers of a microfluidic device, allowed to settle for ~30 minutes, and imaged in every fluorescence color channel with microscopy specifications as described in Methods. Images were obtained from ten

biological replicates (*i.e.*, from overnight cultures prepared on 10 different days) of each single-color control strain.

Cells in images were segmented by thresholding on pixel intensity in phase contrast images, followed by background fluorescence subtraction. Only cells with fluorescence intensity values above 0 a.u. in the true fluorescent color channel were included for calculation of B (mean ± s.d. number of cells included for analysis in a biological replicate were $n = 4{,}765 \pm 1{,}955$; $n = 3{,}246 \pm 1{,}938$; $n = 3{,}367 \pm 1{,}952$ for RFP, YFP, and TFP single-color strains, respectively). All cells across all biological replicates ($n = 10$) were pooled, and the signals of each cell measured in all three fluorescence channels were plotted on pairs of axes each representing a color channel ($n = 47{,}648$; $n = 32{,}458$; $n = 33{,}672$ cells after pooling, for RFP, YFP, and TFP single-color strains, respectively) (Supplementary Fig. 16). Linear least squares regression, assuming an intercept at x = 0 and y = 0, was performed on the pooled fluorescence signal data for the calculation of B (Supplementary Fig. 16):

$$B = \begin{bmatrix} 1 & 9.8 \times 10^{-2} & 7.2 \times 10^{-3} \\ 1.1 \times 10^{-3} & 1 & 4.0 \times 10^{-4} \\ 1.0 \times 10^{-4} & 2.6 \times 10^{-3} & 1 \end{bmatrix}$$

The spectral leakage correction matrix, B, was used to solve for the true fluorescence intensities (t, y, or r) of each cell in microfluidic experiments using Equation S4.

A similar method, with images of single-color strains grown on agarose pads, was used to calculate a different spectral leakage correction matrix, $B_{agarose}$, for the phytoplankton-bacteria co-incubation experiment:

$$B_{agarose} = \begin{bmatrix} 1 & 8.9 \times 10^{-2} & 1.9 \times 10^{-3} \\ 6.0 \times 10^{-4} & 1 & 1.0 \times 10^{-4} \\ -2.0 \times 10^{-4} & 1.1 \times 10^{-2} & 1 \end{bmatrix}$$

1.4. Spectral leakage correction, and thresholding and normalization by constitutive YFP

True fluorescence intensities of each cell were solved for by using the spectral leakage correction matrix, B. The largest correction occurred with teal fluorescence signals, into which YFP leaked significantly (Supplementary Fig. 16). After spectral leakage correction, dim or non-fluorescent (*i.e.*, low metabolic activity or dead) cells were eliminated from further analyses by applying a threshold (50 a.u.) on constitutive YFP fluorescence. The distribution of cellular YFP signals revealed a bimodal distribution: one population with a sharp peak near 0 a.u. containing non-fluorescent or dim cells, and a second, larger population consisting of bright cells. A cutoff YFP intensity of 50 a.u. was applied to all replicate experiments of time-lapse DMSP experiments in microfluidic chips (Supplementary Fig. 6) to eliminate non-fluorescent or dim cells. As a result of thresholding on YFP intensity, 6–33% of recognized cells were eliminated from further analyses.

Finally, RFP and TFP signals of each cell were normalized by YFP fluorescence, which served as a proxy for metabolic activity level and plasmid number. Fluorescence signals in red and teal channels

of each cell were normalized by the mean YFP signal at the corresponding time point and experimental condition (Supplementary Fig. 15).

1.5. Calculation of theoretical 'off' fluorescence intensity

Due to detector noise, a non-zero signal was detected even in the absence of fluorescence expression by cells. Thus, the theoretical 'off' intensity value of cells had to be determined to differentiate absence of fluorescence (*e.g.*, promoters that are turned off) from weak signal intensities (*e.g.*, leaky promoters).

The theoretical 'off' fluorescence intensities were defined as the values of signal leakage detected in the red and teal fluorescence channels while imaging the single-color YFP control strain, after background subtraction, and spectral-leakage correction using *B*. Only YFP cells whose true fluorescence intensities in the yellow channel were above 30 a.u. were included in the analysis.

To calculate the average theoretical 'off' fluorescence intensities, signals in red and teal channels were first averaged across cells in each image, then subsequently averaged across images within a replicate, and normalized by the average YFP intensity of the corresponding replicate. Finally, normalized leakage intensities were averaged across all replicate experiments ($n = 10$). The values (mean ± s.d.) of the theoretical 'off' fluorescence were $-1.6 \pm 0.37 \times 10^{-3}$ a.u. (red channel) and $2.5 \pm 4.1 \times 10^{-3}$ a.u. (teal channel) (Supplementary Fig. 5).

Supplementary Note 2: Interpretation of fluorescence results

2.1. Time-lapse DMSP experiments in microfluidic chips

Results from all replicates of time-lapse DMSP experiments are presented in Supplementary Fig. 6. While the magnitudes of end-point fluorescence intensities (*i.e.*, average fluorescence signals over the final five (~20.4–24 h) time points), and the timepoints of mid-exponential and saturation of fluorescence kinetics curves, were not the same across replicates and strains, other aspects (concentration-dependence of fluorescence intensities and the preservation of slopes of the kinetics curves across concentration) were consistent. The decline in RFP signal after signal saturation may be due to bleaching, plasmid loss, or fluorescent protein degradation. The slight increase in TFP signal in glucose negative control and after signal saturation may be due to a delay in protein folding, or clumping of cells as they reach overgrowth in the experimental chamber, leading to a perceived increase in brightness of cell-containing pixels over time. Due to spectral (*i.e.*, bleaching and effect of cell clumping on signal) and biological (*i.e.*, rate of protein folding and degradation, and effect of plasmid loss) differences between the fluorescent proteins, these phenomena are expected to affect signals detected in red and teal fluorescence channels in different ways. However, these small artefacts did not affect our overall conclusions outlined in the main text.

Temporal evolution of constitutively-expressed YFP fluorescence in all time-lapse DMSP experiments are presented in Supplementary Fig. 15. All experiments were performed with antibiotics (kanamycin) to slow plasmid-loss during experiments, but at a low concentration (10 μg/ml) to minimize interference with biological processes. We observed a 13.4 ± 17.0% (mean ± s.d. of replicate experiments) decay in YFP signals in glucose between 1 h and 10 h of the microfluidic experiments (Supplementary Fig. 15), which may be a predictor of the rate of plasmid-loss in our experiments. Loss of plasmid over the experimental duration may contribute to an underestimation of fluorescence signals reporting *dmdA* and *dddW* expression. However, the relatively small variability in the level of YFP signal decay across replicate experiments suggests that the rate of plasmid-loss is comparable amongst cells on average. Thus, we conclude that comparisons of cell fluorescence relative to each other (e.g., between different concentrations of DMSP, or between *dddW* and *dmdA*) is valid.

2.2. Heterogeneity in DMSP degradation gene expression

Our single-cell approach revealed the emergence of heterogeneity in the DMSP degradation pathway response within a single bacterial strain (*R. pomeroyi* DSS-3). Underlying the population averages were concentration-dependent shifts in the proportions of cells classified in four discrete categories: (1) *dddW* response only, (2) *dmdA* response only, (3) *dddW* + *dmdA* response, and (4) no response (Supplementary Fig. 9).

End-point expression levels were defined as the average fluorescence signals over the last five time points (*i.e.*, ~20.4–24 h) of a time-lapse DMSP experiment performed in observation chambers, and were used as proxies for maximum gene expression levels that *dddW* and *dmdA* reached as a result of exposure to a DMSP concentration (Fig. 2c,d). For DMSP concentrations ranging between 10–50 μM, the mean end-point cleavage (*dddW*) pathway expression level (shown in Fig. 2d) was driven by a population of cells that solely upregulated the *dddW* gene (the '*dddW* response only' population),

which made up nearly half (40.0 ± 21.4% to 44.3 ± 14.9%) of total cells at 10–50 μM (Supplementary Fig. 9g); while the other half of the population maintained baseline expression of the DMSP pathways (the 'no response' population). In contrast, the average end-point demethylation (*dmdA*) pathway expression level (Fig. 2c) was driven by a subpopulation of cells that upregulated both *dmdA* and *dddW* genes (the 'both pathways on' population; Supplementary Fig. 9g). This population grew exponentially between DMSP concentrations of 10 μM (7.0 ± 3.7% of total cells) and 75 μM (28.9 ± 9.3% of total cells), and surpassed all other subpopulations at 250 μM (Supplementary Fig. 9), at which the increase in the maximum gene expression levels with DMSP concentration also dampened (Fig. 2c,d).

Taken together, these results suggest that specific cell subpopulations drove the average increases in *dmdA* and *dddW* gene expression in different DMSP concentration regimes. At the highest DMSP concentrations tested (>100 μM), the majority of cells shifted their metabolic strategy towards upregulating both pathways (*dddW* + *dmdA*), probably in order to capitalize on the abundance of DMSP. However, upregulation of both pathways came at the expense of the ability to respond sensitively to DMSP concentration, as the positive relationship between end-point gene expression levels and concentration dampened above 100 μM (Fig. 2c,d). Finally, we speculate that the shift in the relative expression towards cleavage pathway with increasing DMSP concentration (Fig. 3) is controlled at two distinct levels: (i) at the single cell level (*i.e.*, individual cells increase *dddW* expression levels), as well as (ii) at the population level (*i.e.*, increasing fractions of '*dddW* only' or '*dddW* + *dmdA*' subpopulations with concentration).

Supplementary Note 3: Image analysis for phytoplankton-bacteria co-incubation experiment

3.1. Segmentation of *Breviolum* cells and identification of concentric rings
Phycosphere images were analyzed using a similar MATLAB software as that described for the analysis of observation chamber experiments (Supplementary Note 2). Additional steps and differences in image processing of phycosphere images are described below.

Due to spectral leakage in the red channel by photosynthetic pigments (which masked any RFP signals from bacteria), only signals in the teal (reporting *dmdA* in strain Regular and *dddW* in strain Goofy) and yellow (constitutive YFP expression) channels were quantified, while the red channel was used only to assess the fluorescence signal of *Breviolum* photosynthetic pigments. Images were selected for analysis by screening for bright photosynthetic pigment fluorescence in the red channel (*Breviolum* cells with diffuse, low-intensity pigment fluorescence were excluded from analysis). Furthermore, images with only one phytoplankton cell per field of view (80 μm × 80 μm) were selected. These selection criteria yielded 15 images for strain Regular (reporting *dmdA* with TFP) and 18 images for strain Goofy (reporting *dddW* with TFP), with a combined total of 33 phycosphere images for further analyses.

Segmentation of *Breviolum* cells was achieved through pixel intensity thresholding on photosynthetic pigment signal in the red channel, and approximating *Breviolum* cells as circles. Concentric rings with 20 pixels width (1.6 μm), emanating from the surface of circularly approximated *Breviolum* cells, were used to bin distances within phycospheres. Since the radii of *Breviolum* cells slightly differed across 33 cells (3.3 ± 0.9 μm; mean radius ± s.d.), the distance of concentric rings from the center of *Breviolum* cells varied slightly from image to image. The largest concentric ring (*i.e.*, the farthest distance within the phycosphere) was determined to be the ring before the one that contacted any edge of the image.

3.2. Uneven illumination correction, background fluorescence subtraction, and spectral leakage correction with $B_{agarose}$
Uneven illumination across the field of view was observed at high magnification (100×), probably due to factors related to the light path between the camera and the microscope. To quantitatively correct for uneven illumination, images of agarose pads in the absence of cells were taken in each channel at different stage positions (*n* = 4). At each pixel position, intensities were averaged across the 4 images to create a master 'uneven illumination correction' image. Phycosphere images in teal and yellow channels were corrected by division, at each pixel position, by the master 'uneven illumination correction' image at the corresponding pixel position in corresponding channels. The effect of uneven illumination was small: as an estimate of the magnitude of the maximum correction, the ratio of minimum and maximum intensities in the 'uneven illumination correction' images were 0.81 in teal and 0.87 in yellow.

Background fluorescence intensity was calculated by first roughly identifying *Breviolum*- and bacteria-containing regions by thresholding on pixel intensity in the phase contrast channel (to identify dark/low- and bright/high-intensity pixels), then expanding the region by a distance of 35 pixels. The

negative of this expanded area generated the 'background masks'. Average intensities of background pixels were calculated in teal and yellow fluorescence channels for each phycosphere image, and were subtracted from each non-background pixel.

Phycosphere experiment-specific spectral leakage correction matrix, $B_{agarose}$, was generated by analyzing images of constitutively fluorescent single-color control strains (RFP, YFP, or TFP) grown on agarose pads in the absence of phytoplankton. Single-color bacteria-containing pixels were identified by thresholding on fluorescence intensity, and linear regression on pixel intensities were performed to calculate the spectral leakage correction matrix, $B_{agarose}$. The spectral leakage correction matrix was applied to each pixel of co-incubation images to solve for the true fluorescence intensities in teal and yellow channels.

3.3. Quantification of bacterial *dmdA* and *dddW* gene expression in phycospheres

After background subtraction and spectral leakage correction, pixels containing *R. pomeroyi* cells were identified by thresholding for high YFP fluorescence (proxy for metabolic activity), and for positive teal fluorescence values. Due to differences in magnification and experimental setups, different fluorescence intensity thresholds were chosen for the phycosphere experiments than those applied in the time-lapse DMSP experiments performed in observation chambers. Constitutive YFP was brighter in strain Goofy than in strain Regular, reporting *dddW* and *dmdA* in TFP, respectively, probably due to slight differences in growth phase in each bacterial culture. As such, different thresholds of YFP intensity (>0.08 a.u. for *dmdA* and >0.23 a.u. for *dddW* reporters) were chosen through visual inspection of resulting images, whereby appropriate YFP intensity thresholds accepted cell-containing pixels but excluded background pixels. The YFP intensity threshold values were roughly in the same place in the distribution of pixel intensities for the two reporter strains. Finally, pixels that passed the YFP intensity threshold had to also have positive teal fluorescence values (>0 a.u.) for inclusion in further analyses. Appropriate cell-associated pixel identification and thresholding were visually inspected and confirmed for each image.

Rather than segmenting each bacterial cell, each bacteria-containing pixel was independently quantified. In each phycosphere, only distances at which the area of 20-pixel-width concentric rings contained at least 500 bacteria-associated pixels (equivalent area of a 6.3 μm × 6.3 μm square, or approximately 3 cells) were included for further analyses. Despite this 500-pixel threshold, at least 10 images (out of 15, for *dmdA*) and 14 images (out of 18, for *dddW*) were included for analyses at any given concentric ring distance within the phycosphere. Fluorescence intensity values at each concentric ring were aggregated across images to calculate the average values presented in Fig. 4c,d.

3.4. Calculation of relative expression of *dmdA* and *dddW* in phycospheres

To compare *dmdA* and *dddW* expression levels, TFP intensity (reporting *dmdA* or *dddW*) of each pixel was normalized by its own YFP intensity (Fig. 4e). Cleavage-to-demethylation ratio (*dddW* / *dmdA*) was calculated as the product of the mean normalized TFP intensity of strain Goofy (*dddW*, n = 18) and the mean of the inverse of normalized TFP intensity of strain Regular (*dmdA*, n = 15) at each distance within the phycosphere (Fig. 4f). Error bars represent the variance of the cleavage-to-

demethylation ratio, or the product of cleavage and inverse of demethylation gene expression ($\mu_W \cdot \mu_{\frac{1}{A}}$), and was calculated with the following equation at each distance within the phycosphere:

$$Var\left(\mu_W \cdot \mu_{\frac{1}{A}}\right) = \sigma^2{}_W \cdot \sigma^2{}_{\frac{1}{\mathrm{A}}} + \sigma^2{}_W \cdot \mu^2{}_{\frac{1}{\mathrm{A}}} + \sigma^2{}_{\frac{1}{A}} \cdot \mu^2{}_W \qquad \text{(Eq. S6)}$$

where μ_W and σ_W represent the mean and standard deviation of *dddW* expression level at a concentric ring; and $\mu_{\frac{1}{A}}$ and $\sigma_{\frac{1}{A}}$ are the mean and standard deviation of the inverse of *dmdA* expression levels at a concentric ring.

Supplementary Note 4: Model of DMSP concentrations in the phycosphere

The microenvironment surrounding a unicellular phytoplankton, the 'phycosphere', is often characterized by a concentration gradient of exudates and dissolved organic matter (DOM)[7]. Exudates include DMSP, which comprises up to 10% of fixed carbon in some phytoplankton species[8]. Here, we predict the concentration of exuded DMSP in the phycosphere as a function of the leakage rate, *L*, and its molecular diffusivity, *D* (Supplementary Fig. 13).

Experimentally, we simulated a phycosphere by seeding *R. pomeroyi* fluorescent reporter bacteria with *Breviolum* phytoplankton cells on agarose pads, which preserved the spatial arrangement of co-incubated organisms (Methods). We assumed a 30% reduction in diffusivity of DMSP in agarose ($D_{agarose}$) compared to solution ($D_{solution}$ = 0.5 × 10^{-5} $cm^2\,s^{-1}$)[9]. A similar percentage of diffusivity reduction was observed for sucrose in agarose in a previous study[10]. The thickness of agarose (~0.5 mm) was much larger (150×) than the average radius of *Breviolum* cells (3.3 μm); thus, a 3D diffusion model was used to describe the concentration of DMSP in agarose. However, since the phytoplankton cell was immobilized with agarose on one side, diffusion may be more accurately approximated on a half-volume around the cell, instead of its entire volume. Thus, the effective leakage rate would be double of that expected (2*L*). Taken together, the concentration of DMSP in agarose at distance *r* from the center of a phytoplankton cell is modeled as:

$$C_r = \frac{2L}{4\pi D_{agarose} r} \qquad \text{(Eq. S7)}$$

with *L*, the constant exudation rate (mol/s) of DMSP, defined as:

$$L = \mu\theta f \qquad \text{(Eq. S8)}$$

where μ is the doubling rate of the *Breviolum* cell, θ is the amount of intracellular DMSP if there were no leakage ($\mu\theta$ is thus the rate of production of DMSP by the cell), and f is the fraction of the production rate that is leaked.

The actual, stable quantity of intracellular DMSP that is maintained by the cell, ϕ, is defined with the following relationship with θ and f:

$$\phi = \theta(1-f) \qquad \text{(Eq. S9)}$$

Thus, *L*, the constant exudation rate (mol/s) of DMSP, is described as:

$$L = \frac{\mu\phi f}{1-f} \qquad \text{(Eq. S10)}$$

Breviolum cells in our experiment were most likely under physiological stress, or undergoing senescence, due to immobilization on an agarose pad, proximity to high numbers of bacteria, and

exposure to vigorous shaking of cell cultures prior to co-incubation; stress and shaking have been shown to increase intracellular DMSP and exudation rate[11,12]. However, all *Breviolum* cells included in our image analyses maintained high fluorescence signal from photosynthetic pigments to ensure viable phycospheres at the time of imaging. We considered the leakage fraction (f) to be constant and not dependent on cell size; however, in reality, leakage fraction may be expected to decrease with increasing cell size, though little information is available on this issue.

Up to 50% of all organic materials produced can be released by phytoplankton[13,14], and a survey of intracellular DMSP measurements of 55 samples of Symbiodiniaceae found a range of 0.021–3.831 pmol DMSP per cell[15]. In this model, we assumed a doubling rate (μ) of 1 day^{-1} or 86,400 s^{-1}, a leakage fraction (f) of 1/9 (~11%), and an intracellular content (ϕ) of 1 pmol per cell for a small, spherical phytoplankton (mean radius of *Breviolum* cells in our experiment was 3.3 μm), in the absence of fluid flow.

Two further cases, in which μ, ϕ, $\frac{f}{1-f}$, or 1/$D_{agarose}$ by themselves, or the product of these four terms overall, is 3-fold greater or 3-fold smaller, are calculated and shown as upper and lower boundaries compared to the baseline case scenario (the edges of the shaded region in Supplementary Fig. 13). The former case (*i.e.*, upper boundary) represents the scenario in which parameters are as in the baseline case, but leakage or intracellular DMSP content is high (as is the case for stressed cells[11]; in our model, $f = 0.25$ or $\phi = 3$ pmol per cell) or diffusivity is low (*e.g.*, agarose reduces diffusivity by more than 30% of $D_{solution}$; in our model, $D_{agarose} = 0.12 \times 10^{-5}$ cm^2 s^{-1}). The same reasoning applies to the lower boundary. Parameters for the baseline case were chosen to represent realistic values, and the two further curves thus provide a measure of possible DMSP concentration ranges in a phycosphere under different conditions.

Supplementary References

1. Cox III, R. S., Dunlop, M. J. & Elowitz, M. B. A synthetic three-color scaffold for monitoring genetic regulation and noise. *J. Biol. Eng.* **4**, 10 (2010).
2. Kovach, M. E. *et al.* Four new derivatives of the broad-host-range cloning vector pBBR1MCS, carrying different antibiotic-resistance cassettes. *Gene* **166**, 175–176 (1995).
3. Persat, A., Stone, H. A. & Gitai, Z. The curved shape of *Caulobacter crescentus* enhances surface colonization in flow. *Nat. Commun.* **5**, 3824 (2014).
4. Keen, N. T., Tamaki, S., Kobayashi, D. & Trollinger, D. Improved broad-host-range plasmids for DNA cloning in Gram-negative bacteria. *Gene* **70**, 191–197 (1988).
5. Reisch, C. R. *et al.* Novel pathway for assimilation of dimethylsulphoniopropionate widespread in marine bacteria. *Nature* **473**, 208–211 (2011).
6. Kessler, B., de Lorenzo, V. & Timmis, K. N. A general system to integrate *lacZ* fusions into the chromosomes of Gram-negative eubacteria: regulation of the *Pm* promoter of the *TOL* plasmid studied with all controlling elements in monocopy. *Mol. Genet. Genomics* **233**, 293–301 (1992).
7. Mitchell, J. G., Okubo, A. & Fuhrman, J. A. Microzones surrounding phytoplankton form the basis for a stratified marine microbial ecosystem. *Nature* **316**, 58–59 (1985).
8. Matrai, P. A. & Keller, M. D. Total organic sulfur and dimethylsulfoniopropionate in marine phytoplankton: intracellular variations. *Mar. Biol.* **119**, 61–68 (1994).
9. Spiese, C. E. Determination of the diffusion constants of dimethylsulfide and dimethylsulfoniopropionate by diffusion-ordered nuclear magnetic resonance spectroscopy. *Mar. Chem.* **207**, 77–83 (2018).
10. Lundberg, P. & Kuchel, P. W. Diffusion of solutes in agarose and alginate gels: ^{1}H and ^{23}Na PFGSE and ^{23}Na TQF NMR studies. *Magn. Reson. Med.* **37**, 44–52 (1997).
11. Stefels, J. Physiological aspects of the production and conversion of DMSP in marine algae and higher plants. in *Journal of Sea Research* **43**, 183–197 (2000).
12. Berdalet, E., Llaveria, G. & Simó, R. Modulation of dimethylsulfoniopropionate (DMSP) concentration in an *Alexandrium minutum* (Dinophyceae) culture by small-scale turbulence: a link to toxin production? *Harmful Algae* **11**, 88–95 (2011).
13. Thornton, D. C. O. Dissolved organic matter (DOM) release by phytoplankton in the contemporary and future ocean. *Eur. J. Phycol.* **49**, 20–46 (2014).
14. Seymour, J. R., Amin, S. A., Raina, J.-B. & Stocker, R. Zooming in on the phycosphere: the ecological interface for phytoplankton-bacteria relationships. *Nat. Microbiol.* **2**, 17065 (2017).
15. Caruana, A. M. N. & Malin, G. The variability in DMSP content and DMSP lyase activity in marine dinoflagellates. *Prog. Oceanogr.* **120**, 410–424 (2014).
