# Supplementary material for: Single-cell bacterial transcription measurements reveal the importance of dimethylsulfoniopropionate (DMSP) hotspots in ocean sulfur cycling
Source: Nat Commun. 2020 Apr 23;11:1942. doi: 10.1038/s41467-020-15693-z (PMC7181598; doi:10.1038/s41467-020-15693-z)
Supplement: Supplementary file 3 — Reporting Summary [file 41467_2020_15693_MOESM3_ESM.pdf]

nature research

Corresponding author(s): Jean-Baptiste Raina & Roman Stocker

Last updated by author(s): Mar 9, 2020

Reporting Summary

Nature Research wishes to improve the reproducibility of the work that we publish. This form provides structure for consistency and transparency in reporting. For further information on Nature Research policies, see Authors & Referees and the Editorial Policy Checklist.

Statistics

For all statistical analyses, confirm that the following items are present in the figure legend, table legend, main text, or Methods section.

n/a	Confirmed	
☐	☒	The exact sample size (*n*) for each experimental group/condition, given as a discrete number and unit of measurement
☐	☒	A statement on whether measurements were taken from distinct samples or whether the same sample was measured repeatedly
☐	☒	The statistical test(s) used AND whether they are one- or two-sided<br>*Only common tests should be described solely by name; describe more complex techniques in the Methods section.*
☒	☐	A description of all covariates tested
☒	☐	A description of any assumptions or corrections, such as tests of normality and adjustment for multiple comparisons
☐	☒	A full description of the statistical parameters including central tendency (e.g. means) or other basic estimates (e.g. regression coefficient) AND variation (e.g. standard deviation) or associated estimates of uncertainty (e.g. confidence intervals)
☐	☒	For null hypothesis testing, the test statistic (e.g. *F*, *t*, *r*) with confidence intervals, effect sizes, degrees of freedom and *P* value noted<br>*Give P values as exact values whenever suitable.*
☒	☐	For Bayesian analysis, information on the choice of priors and Markov chain Monte Carlo settings
☒	☐	For hierarchical and complex designs, identification of the appropriate level for tests and full reporting of outcomes
☒	☐	Estimates of effect sizes (e.g. Cohen's *d*, Pearson's *r*), indicating how they were calculated

Our web collection on statistics for biologists contains articles on many of the points above.

Software and code

Policy information about availability of computer code

Data collection	NIS-Elements software was used for microscopy data collection.
Data analysis	MATLAB (versions 2016–2019) was used for data analysis.

For manuscripts utilizing custom algorithms or software that are central to the research but not yet described in published literature, software must be made available to editors/reviewers. We strongly encourage code deposition in a community repository (e.g. GitHub). See the Nature Research guidelines for submitting code & software for further information.

Data

Policy information about availability of data

All manuscripts must include a data availability statement. This statement should provide the following information, where applicable:

- Accession codes, unique identifiers, or web links for publicly available datasets
- A list of figures that have associated raw data
- A description of any restrictions on data availability

The data that support the findings of this study are available from the corresponding authors on request (total data size approximately 1 TB).

Field-specific reporting

Please select the one below that is the best fit for your research. If you are not sure, read the appropriate sections before making your selection.

☐ Life sciences ☐ Behavioural & social sciences ☒ Ecological, evolutionary & environmental sciences

For a reference copy of the document with all sections, see nature.com/documents/nr-reporting-summary-flat.pdf

Ecological, evolutionary & environmental sciences study design

All studies must disclose on these points even when the disclosure is negative.

Study description	In this study, a model marine microorganism (Ruegeria pomeroyi DSS-3) was genetically engineered to visually report the expression of dimethylsulfoniopropionate (DMSP) degradation genes. Unless otherwise noted, three replicate experiments, each with hundreds to thousands of cells, were performed.
Research sample	Ruegeria pomeroyi DSS-3 strains transformed with engineered plasmids containing 1–3 promoter-fluorescent protein fusions.
Sampling strategy	For each experiment, a single colony was picked from a culture plate to inoculate overnight liquid cultures (unless otherwise noted). Cells were prepared for experimentation at the same growth phase (OD) for each experiment. In each observation chamber of a microfluidic chip, microscopy fields of views were manually selected with roughly equal spacing for time-lapse observation.
Data collection	We used phase contrast and fluorescence microscopy to capture time-resolved images.
Timing and spatial scale	Images were captured every 45 minutes for 24 hours. Images were acquired with an electron multiplying CCD (EMCCD) camera (iXon3 885; Andor Technology) (1004 × 1002 pixels at 8 μm/pixel), unless otherwise indicated.
Data exclusions	N/A
Reproducibility	Unless otherwise indicated, three replicates were generated and confirmed to generate consistent results.
Randomization	N/A
Blinding	N/A

Did the study involve field work? ☐ Yes ☒ No

Reporting for specific materials, systems and methods

We require information from authors about some types of materials, experimental systems and methods used in many studies. Here, indicate whether each material, system or method listed is relevant to your study. If you are not sure if a list item applies to your research, read the appropriate section before selecting a response.

Materials & experimental systems

n/a	Involved in the study	
☒	☐	Antibodies
☒	☐	Eukaryotic cell lines
☒	☐	Palaeontology
☒	☐	Animals and other organisms
☒	☐	Human research participants
☒	☐	Clinical data

Methods

n/a	Involved in the study	
☒	☐	ChIP-seq
☒	☐	Flow cytometry
☒	☐	MRI-based neuroimaging
